# Supplementary figures and images for: Predominance of positive epistasis among drug resistance-associated mutations in HIV-1 protease
Source: PLoS Genet. 2020 Oct 21;16(10):e1009009. doi: 10.1371/journal.pgen.1009009 (PMC7605711; doi:10.1371/journal.pgen.1009009)

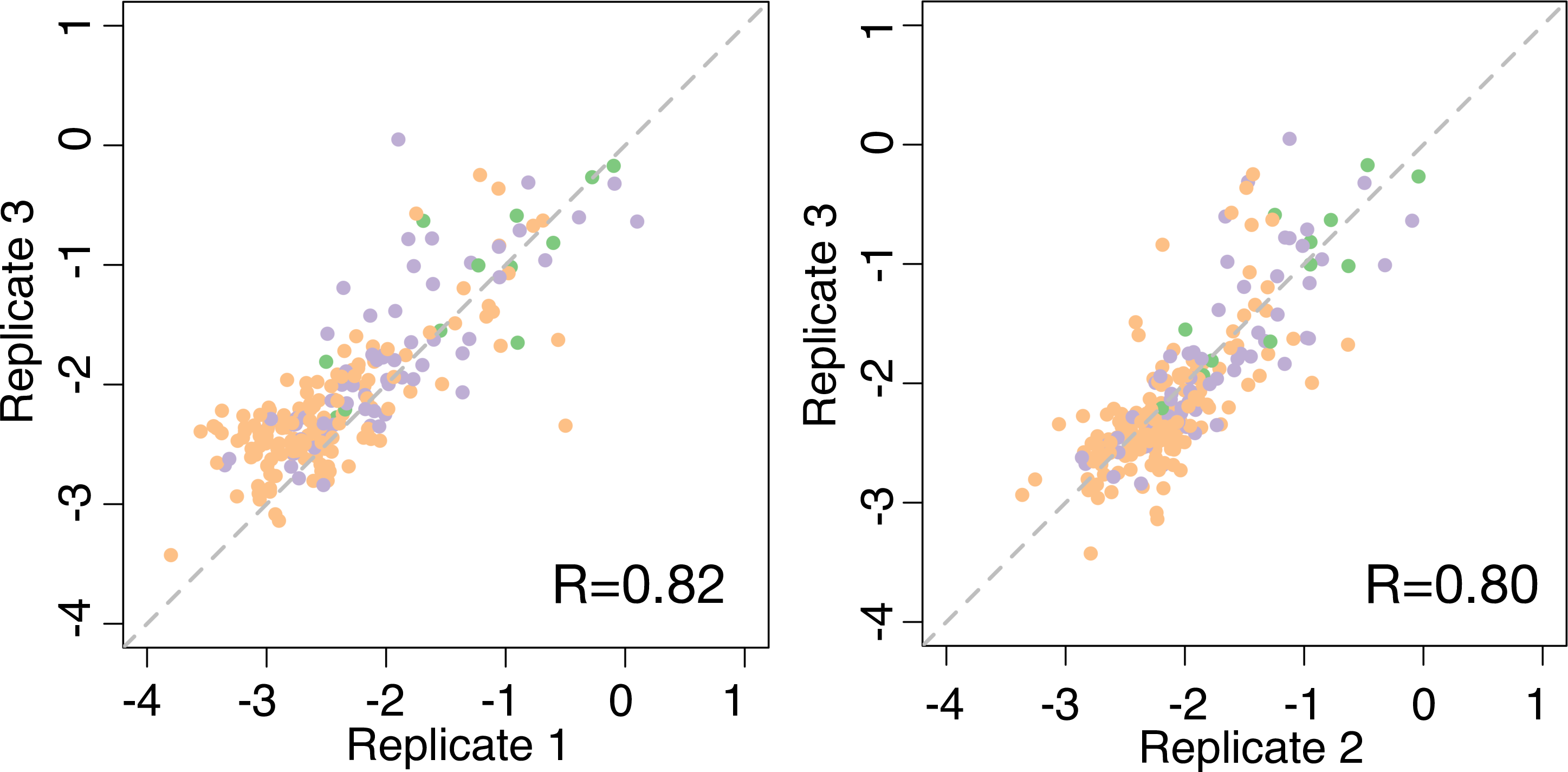

Supplement: S1 Fig — All single mutants, double mutants and triple mutants are shown. R stands for Pearson correlation coefficient. (TIFF) [file pgen.1009009.s001.tiff]

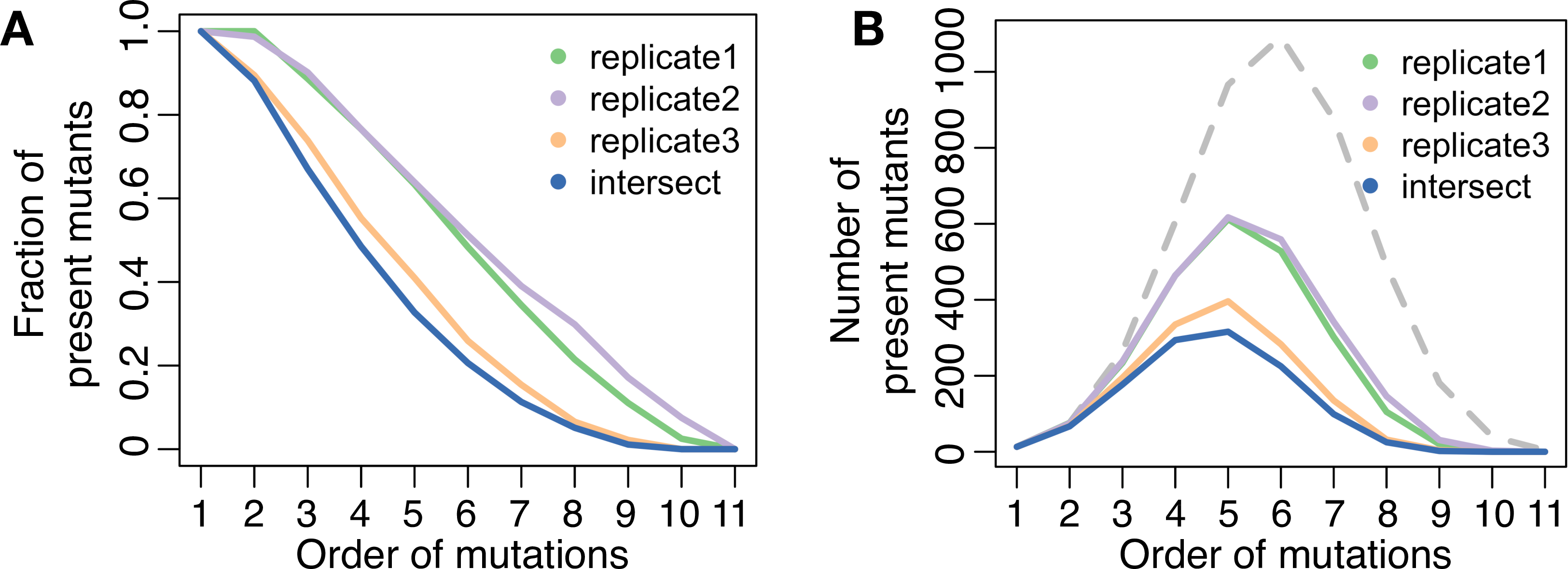

Supplement: S2 Fig — (A) Fraction of expected protease mutants in each transfection virus library. (B) Number of mutant in each transfection virus library. Dashed line represents the number of all possible combinations of mutations. (TIFF) [file pgen.1009009.s002.tiff]

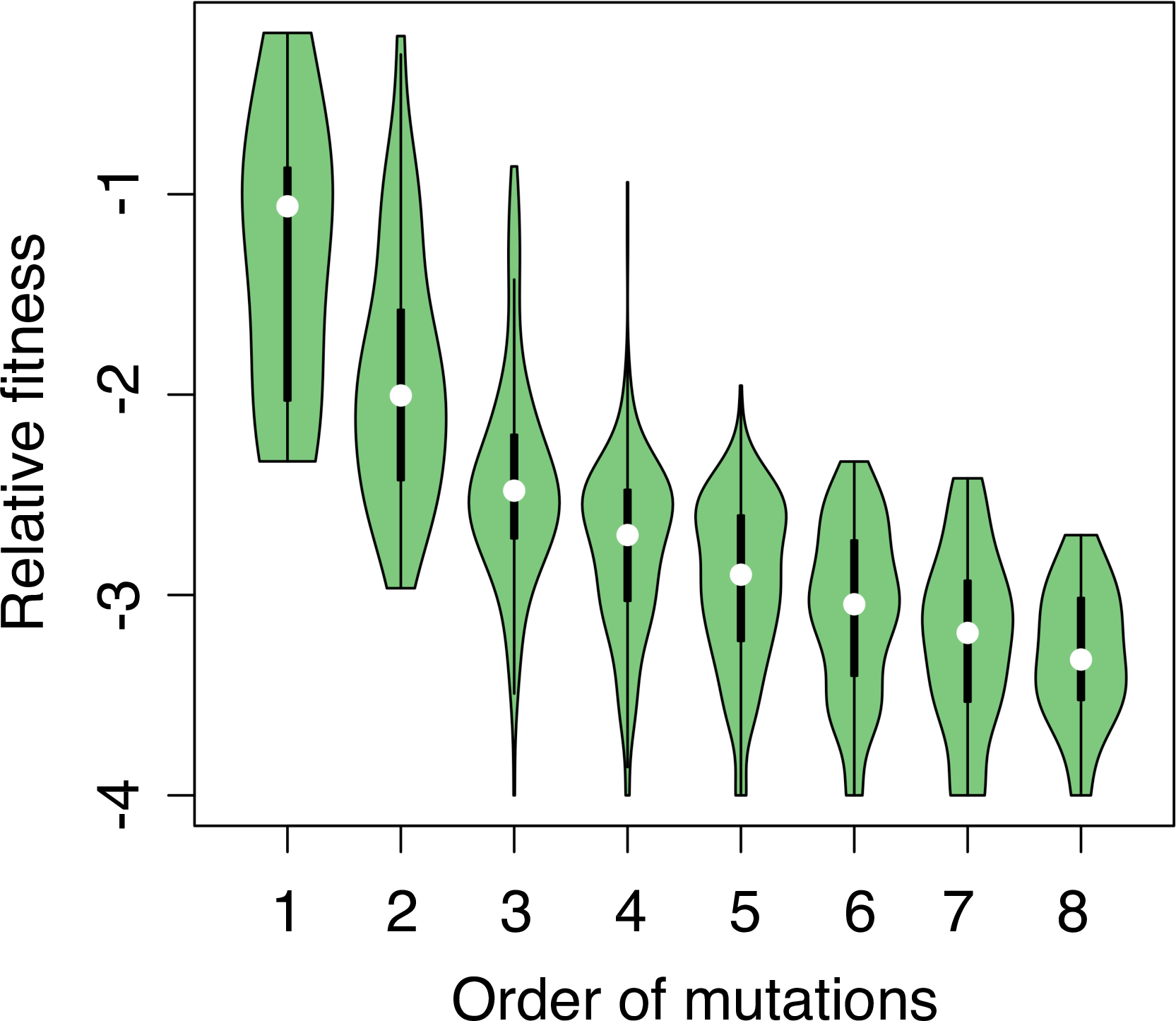

Supplement: S3 Fig — (TIFF) [file pgen.1009009.s003.tiff]

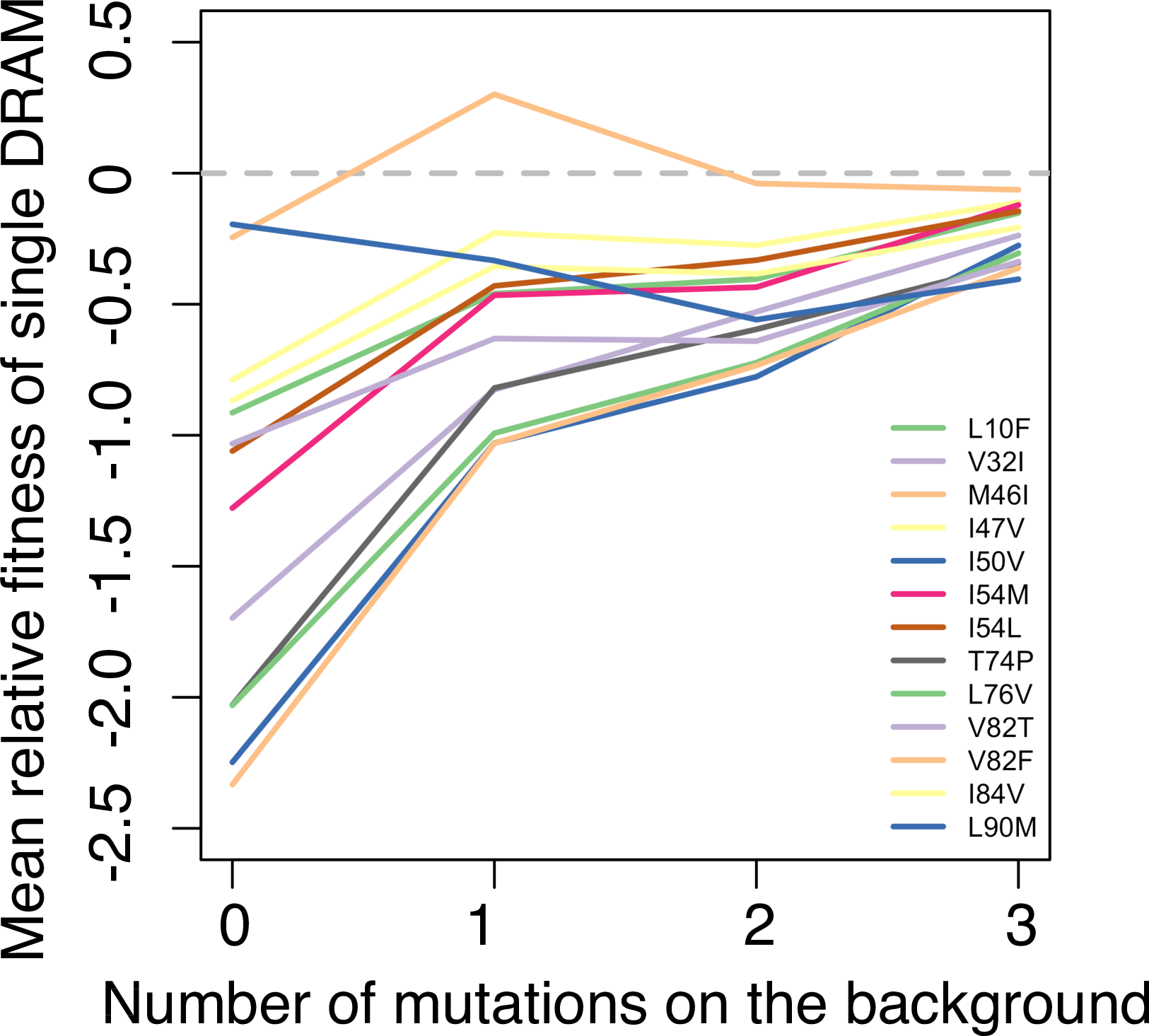

Supplement: S4 Fig — (TIFF) [file pgen.1009009.s004.tiff]

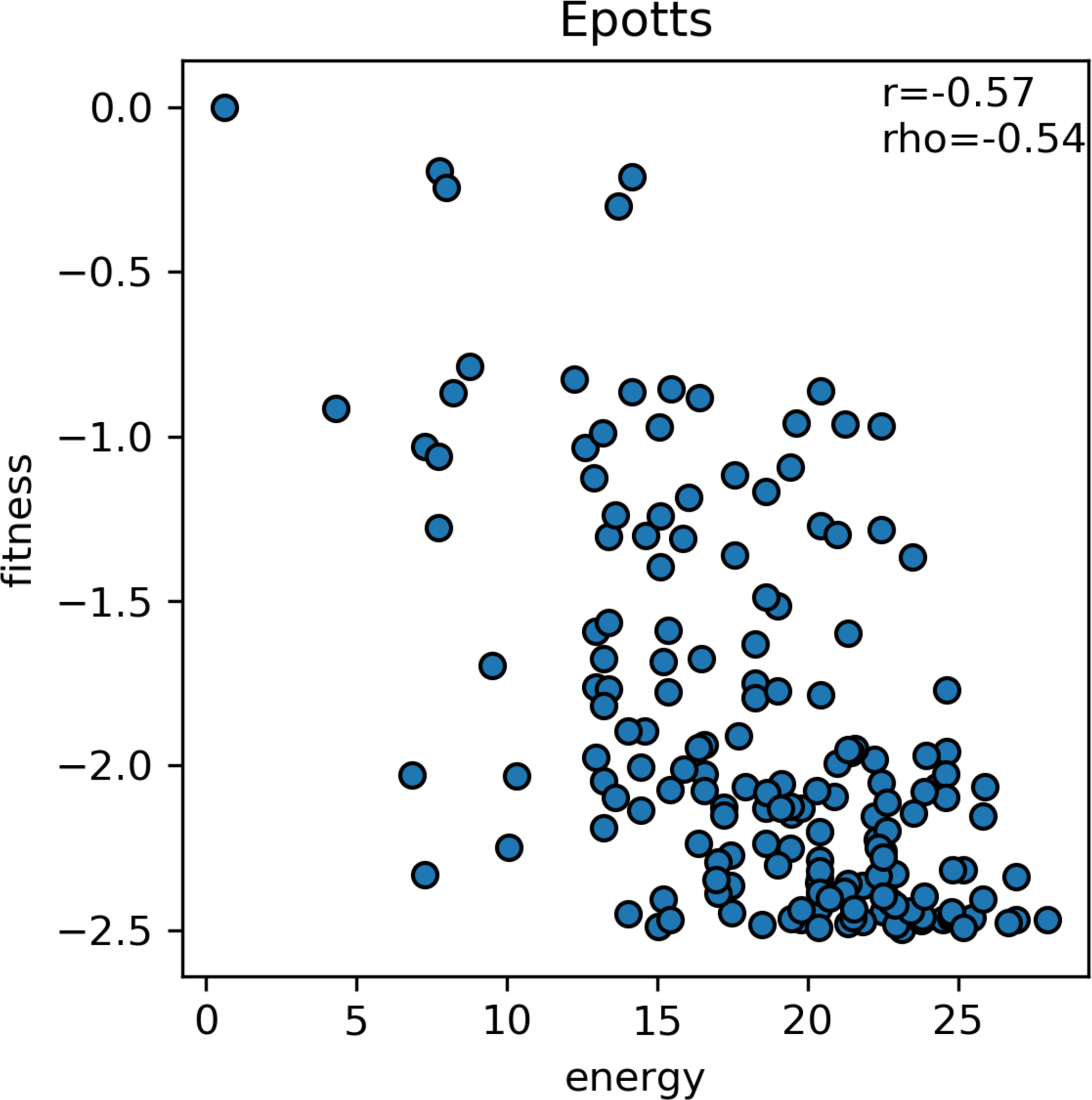

Supplement: S5 Fig — Mutants with relative fitness higher than −2.5 and numbers of mutations lower than 4 is shown. The Pearson’s correlation coefficient is −0.57. The Spearman’s correlation coefficient is −0.54. (TIFF) [file pgen.1009009.s005.tiff]

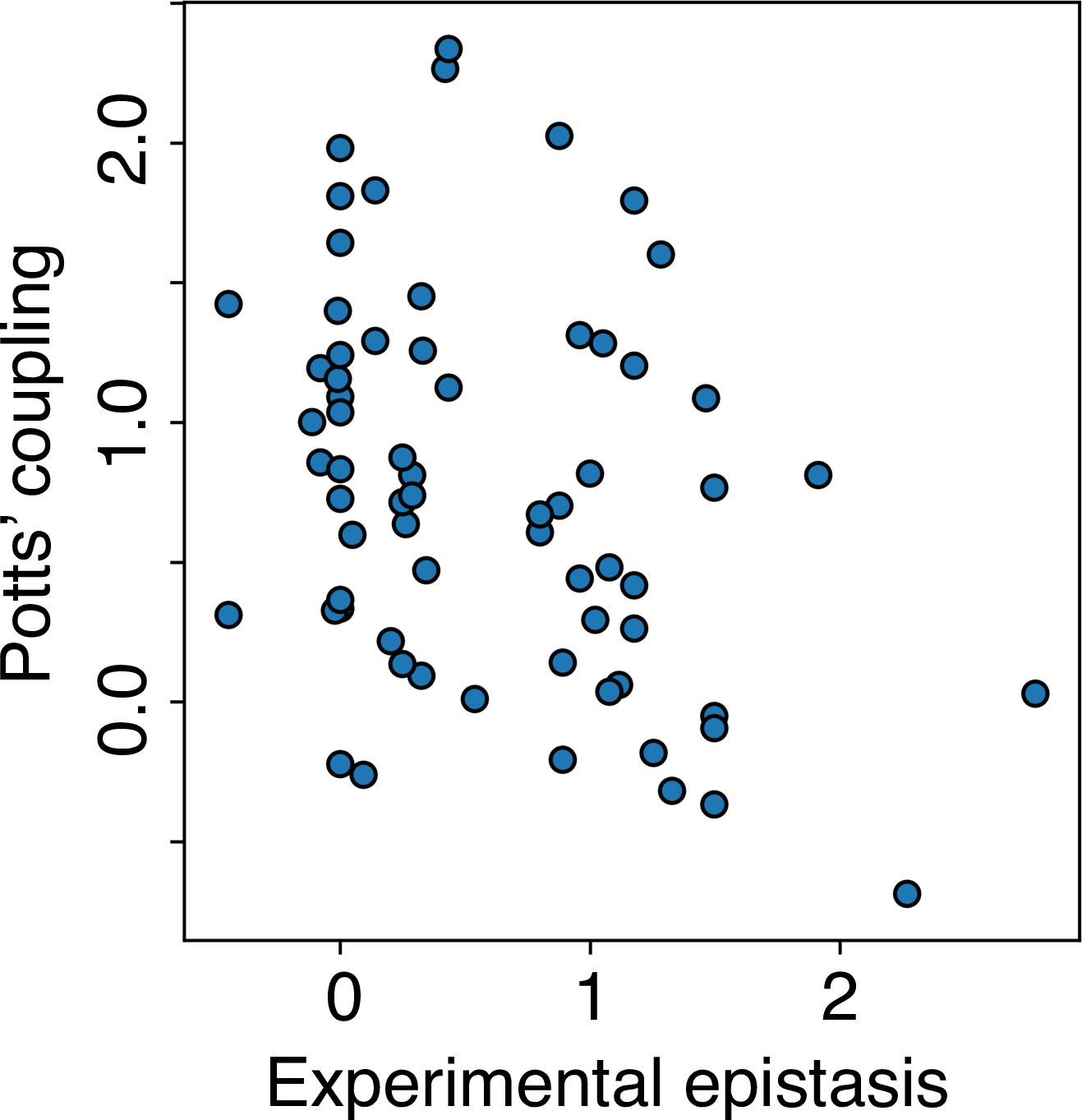

Supplement: S6 Fig — The pairwise epistasis between all RAMs in our library was compared with Potts’ coupling parameters. The Spearman’s correlation coefficient is −0.33. The p value for the Spearman’s correlation coefficient is 6.8 × 10^−3^. (TIFF) [file pgen.1009009.s006.tiff]

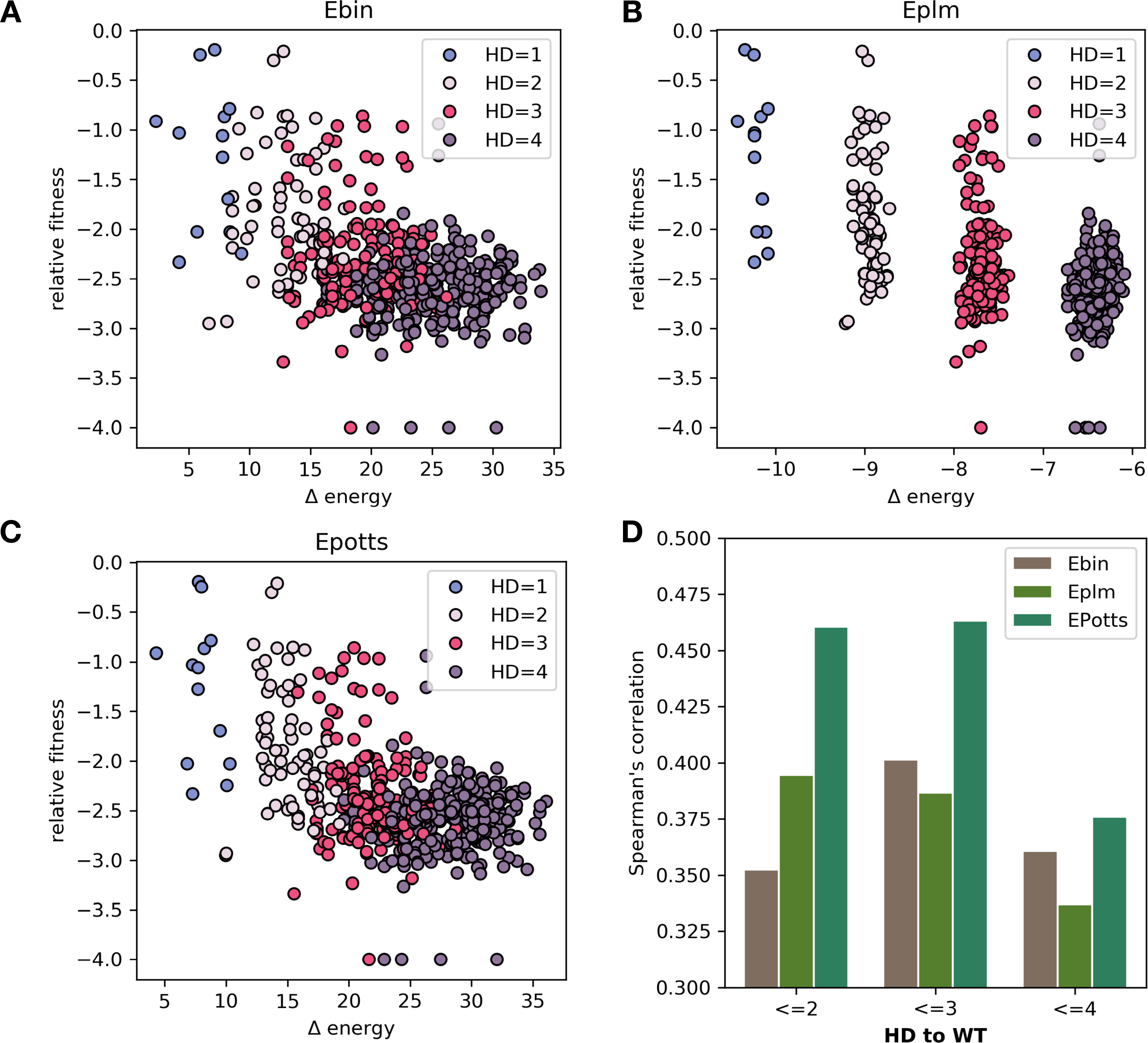

Supplement: S7 Fig — (A, B & C)The correlation between relative fitness with (A, bin) binary (Ising) model inferred via ACE, (B, plm) the Potts model inferred via pseudo-likelihood maximization, or (C, potts) the Potts model inferred via ACE. (D) Spearman’s correlation coefficients for different models. Mutants were classified according to their HD to wild-type. HD, hamming distance. (TIFF) [file pgen.1009009.s007.tiff]

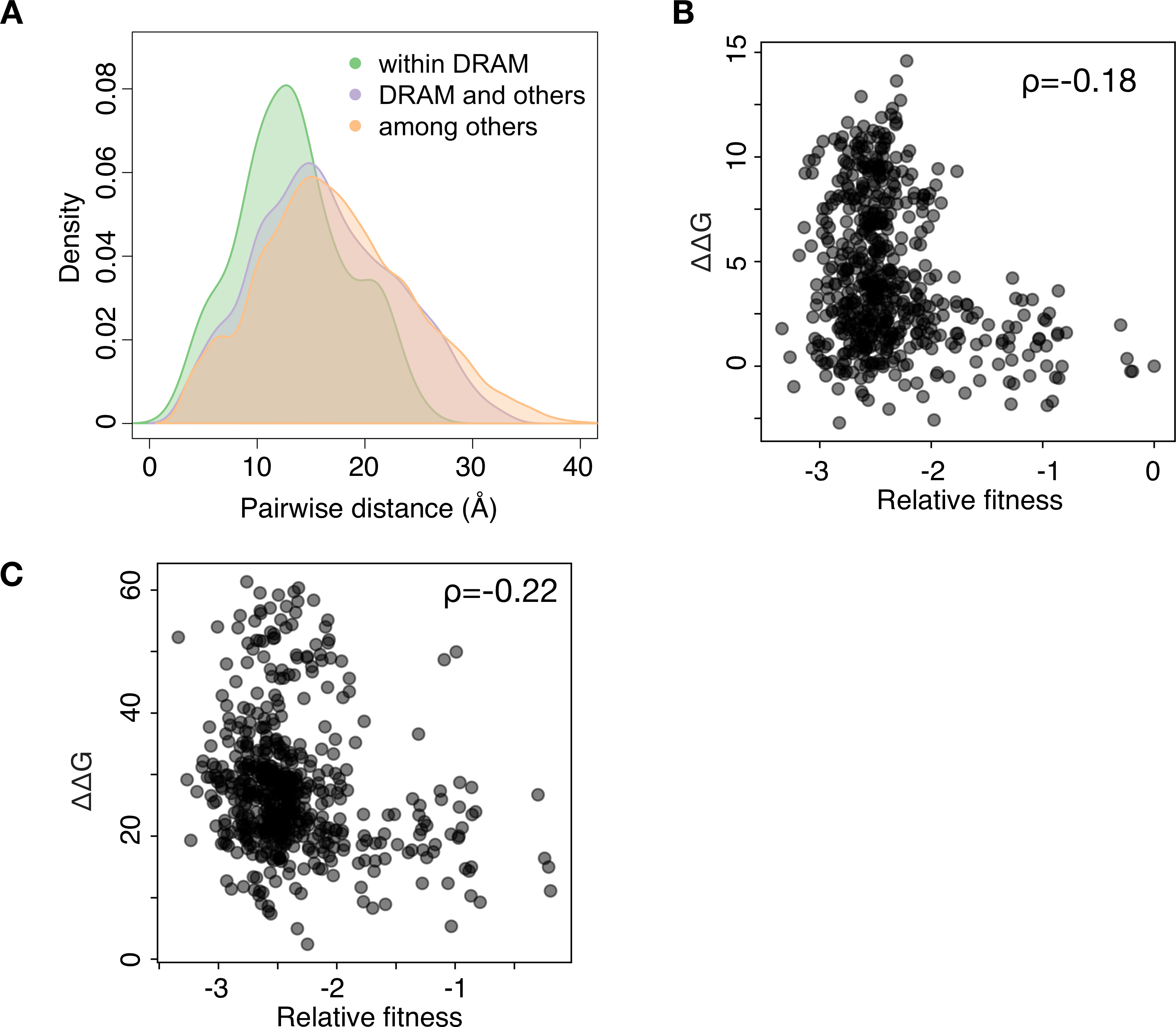

Supplement: S8 Fig — (A) Distribution of pairwise distance among resistance associated residues and other residues. The distance between the C-α of two residues was shown. (B & C) Correlation between mutants’ relative fitness and protein stability (ΔΔG). ΔΔG is predicted by FoldX (B) or Rosetta (C). The correlation coefficients were calculated for mutants with lower than 5 mutations. ρ stands for Spearman’s correlation coefficient. (TIFF) [file pgen.1009009.s008.tiff]
