# Supplementary material for: Predominance of positive epistasis among drug resistance-associated mutations in HIV-1 protease
Source: PLoS Genet. 2020 Oct 21;16(10):e1009009. doi: 10.1371/journal.pgen.1009009 (PMC7605711; doi:10.1371/journal.pgen.1009009)
Supplement: S1 Table — (PDF) [file pgen.1009009.s009.pdf]

Supplementary Table1: Relative fitness of all mutants in this research.

mutants	relative fitness
WT	0
L90M	-0.1947913
M46I_I54M	-0.210517798
M46I	-0.245238013
M46I_I84V	-0.301304513
I47V	-0.788208628
L10F_I47V	-0.826837086
V32I_M46I	-0.856170224
M46I_I47V_I84V	-0.86114999
M46I_I54L	-0.863881775
I84V	-0.867699991
V32I_I47V	-0.883212633
M46I_I47V_I50V_I54M_L76V_V82T_L90M	-0.883212633
L10F	-0.913817233
V32I_M46I_I47V_I54L	-0.940149914
L10F_V32I_I47V	-0.960941838
V32I_M46I_I47V	-0.963964395
V32I_I47V_I54L	-0.968889717
M46I_I47V	-0.972647054
M46I_T74P	-0.990364837
V82T	-1.032011112
I84V_L90M	-1.033638197
I54L	-1.060154581
M46I_I54L_T74P	-1.092675437
M46I_I54M_L90M	-1.116853449
M46I_L90M	-1.126921682
M46I_I54L_V82T	-1.167354998
I47V_I84V	-1.184826166
V32I_I47V_I50V_L76V_V82T_I84V_L90M	-1.184826166
M46I_V82T	-1.240289455
I47V_I54L	-1.240961403
V32I_M46I_I47V_I54M	-1.259988096
M46I_I47V_I54L	-1.272026076
I54M	-1.277808526
V32I_I47V_I54M	-1.284242454
V32I_I47V_L90M	-1.298435
I47V_L90M	-1.300364322
I54L_L90M	-1.305172205
M46I_I84V_L90M	-1.309589752
M46I_I54L_L90M	-1.360177638
V32I_I47V_I84V	-1.365355229

I47V_I54M	-1.395415771
M46I_I54M_V82T	-1.487544746
M46I_I54M_I84V	-1.513918186
I54M_L90M	-1.565735772
V82T_I84V	-1.588215883
V82T_L90M	-1.593221873
V32I_M46I_I54L	-1.598126276
V82T_I84V_L90M	-1.630568215
I54L_V82T	-1.675293989
M46I_I50V	-1.675709393
I47V_V82T	-1.684426388
V32I	-1.697353755
V82F_I84V_L90M	-1.748461422
V82F_L90M	-1.761803237
T74P_V82T	-1.768231494
L76V_V82T_I84V	-1.769719912
M46I_I54L_I84V	-1.774813901
V82F_I84V	-1.775726323
M46I_I47V_I54M	-1.784561117
I47V_I50V	-1.794096798
I54M_V82F	-1.816938041
V32I_M46I_I47V_L90M	-1.843399904
I54L_T74P	-1.893931642
T74P_I84V	-1.894488548
M46I_L76V	-1.91093759
V32I_I47V_I54L_L90M	-1.91882379
V32I_I47V_I54M_T74P_V82F	-1.934522891
L76V_V82T	-1.937497084
V32I_I54L	-1.944788899
T74P_V82F_I84V	-1.947423102
V32I_M46I_I54M	-1.953058489
V32I_M46I_I47V_I54L_L90M	-1.954856554
L76V_V82F_I84V	-1.957625437
V32I_I50V_I54M_L76V_V82T	-1.95818681
I47V_I54L_T74P	-1.96049561
V32I_M46I_I47V_I84V	-1.968577704
V32I_M46I_I50V	-1.970550543
V32I_I47V_I54L_T74P_I84V	-1.972110531
L10F_V32I	-1.974487084
L76V_V82T_L90M	-1.980235117
M46I_I47V_I54L_T74P_L76V	-1.983374356
V32I_M46I_I84V	-1.9946296
V32I_I47V_T74P_V82F_I84V	-2.000519279

I54M_I84V	-2.004383013
I50V_T74P	-2.011847712
L76V_V82F_I84V_L90M	-2.011943547
M46I_I47V_I50V_I54M	-2.01525405
I47V_I50V_I54M	-2.024613231
L76V_V82F	-2.026035294
T74P	-2.029118972
L76V	-2.030416901
M46I_I50V_I54L_L76V_L90M	-2.030737477
I54M_V82T	-2.046764597
I47V_T74P_I84V	-2.052456726
I47V_I84V_L90M	-2.05493939
V32I_I47V_I50V	-2.063303705
T74P_L76V_I84V	-2.065125421
L76V_I84V	-2.065693468
V32I_I47V_T74P_L76V	-2.073184427
V32I_L90M	-2.074073617
V32I_M46I_T74P	-2.075673277
M46I_I50V_I54M_V82F_I84V_L90M	-2.076494967
T74P_L76V	-2.077428745
M46I_I54L_L76V	-2.078195193
M46I_I47V_L90M	-2.081019043
V32I_I47V_I50V_I54L_L76V_V82T	-2.087512327
M46I_I47V_I50V_I54L	-2.090353645
I54L_T74P_I84V	-2.09563053
M46I_V82F	-2.096463289
I47V_I50V_I54L	-2.097764705
M46I_I47V_I84V_L90M	-2.099780257
M46I_I50V_I54M	-2.112316444
I47V_I50V_I54M_L76V_I84V	-2.113316378
V32I_M46I_I47V_I50V	-2.118612048
V32I_M46I_I50V_I54M	-2.121769552
M46I_I54M_T74P	-2.124972748
I50V_I54M	-2.125932254
M46I_V82T_I84V	-2.128404194
V32I_M46I_L90M	-2.128509809
V32I_M46I_I47V_I50V_I54L	-2.129531748
M46I_I54M_V82F	-2.130902562
I54L_I84V	-2.135465128
M46I_I47V_I54L_T74P	-2.140006582
M46I_I47V_I50V	-2.144028864
I47V_I54L_L90M	-2.148680321
I50V_I54L	-2.151917199

L76V_V82T_I84V_L90M	-2.152845028
V32I_I50V_I54L	-2.153086767
V32I_I47V_I54M_T74P_L76V	-2.154680046
L76V_V82F_L90M	-2.154717874
I47V_I50V_I54M_L76V_V82F	-2.160834114
T74P_L76V_V82F_I84V	-2.175853194
V32I_M46I_I47V_I50V_I54M	-2.184572229
V32I_I54L_L76V_V82T_L90M	-2.189795396
I54L_V82F	-2.190232595
I54L_T74P_L76V_V82F	-2.195222292
M46I_I47V_I50V_L76V_I84V_L90M	-2.195222292
M46I_I50V_I54L	-2.198939091
V32I_I50V_I54L_L76V_V82F_I84V_L90M	-2.199340714
I54M_V82T_I84V	-2.200659632
M46I_I50V_I54L_L76V_V82F_I84V_L90M	-2.205589664
M46I_I47V_I50V_I54M_V82T_I84V	-2.207218347
V32I_I47V_T74P_V82T	-2.207973642
I47V_I50V_I54M_L76V_V82T_L90M	-2.208680741
V32I_T74P_L76V_V82F	-2.211487126
V32I_M46I_L76V_V82F_L90M	-2.21426045
V32I_I47V_I50V_I54M	-2.215570595
M46I_I54L_L76V_I84V	-2.218914861
M46I_I50V_I54L_L76V	-2.220901896
L76V_I84V_L90M	-2.225027794
V32I_M46I_I50V_I54L	-2.234284901
V32I_I54M	-2.236784796
M46I_I54L_V82F	-2.237345333
M46I_I47V_I54M_V82F	-2.23933252
M46I_I47V_I50V_I54M_I84V	-2.243136907
M46I_I47V_I54L_L90M	-2.247681386
I50V	-2.248032685
V32I_I50V_I54M_L76V_V82T_I84V	-2.249494475
V32I_I54M_T74P	-2.250094164
V32I_I47V_I50V_I54L	-2.250888464
I54L_T74P_V82F	-2.250930956
I47V_L76V_V82T_I84V_L90M	-2.252586226
I47V_I54M_V82T_I84V	-2.257536251
I54L_L76V_V82F	-2.260651203
M46I_I47V_I50V_I54M_L76V_V82T_I84V	-2.261510143
V32I_M46I_I50V_I54M_V82T_I84V_L90M	-2.261525606
V32I_M46I_I47V_I54M_L76V_V82T_I84V_L90M	-2.263581611
I47V_I50V_I54M_L76V_V82F_I84V_L90M	-2.265263792
M46I_I47V_I50V_V82T	-2.266425396

V32I_M46I_I47V_I50V_I54M_V82T_I84V	-2.268739886
M46I_I47V_I54L_L76V_V82T_L90M	-2.268976643
V32I_M46I_L76V_I84V_L90M	-2.268976643
V32I_M46I_I47V_I50V_I54M_L76V_I84V_L90M	-2.270464986
I54M_L76V	-2.271981867
M46I_I47V_I54M_L76V_V82F_I84V	-2.274143518
I47V_I50V_I54L_L76V	-2.275724356
V32I_I50V_I54M_V82T	-2.275898962
V32I_M46I_I50V_I54M_L76V_V82F	-2.276697154
V32I_I47V_I50V_I54M_L76V_I84V_L90M	-2.27730441
V32I_I50V_T74P_V82F	-2.27730441
V32I_I47V_V82F	-2.277491332
M46I_I54M_T74P_V82F_I84V	-2.279334536
V32I_M46I_I47V_I50V_I54L_V82F	-2.280390425
V32I_I47V_I50V_I54M_V82F_I84V	-2.280471435
V32I_M46I_I47V_I54M_L76V_V82T_I84V	-2.282337614
I47V_I50V_I54L_I84V_L90M	-2.283543603
V32I_M46I_I54M_L76V_V82F_L90M	-2.28477091
V32I_M46I_I47V_I50V_I54L_L76V_V82F_L90M	-2.28477091
I54M_V82F_I84V	-2.286755528
M46I_I47V_I54L_T74P_V82F_I84V	-2.289125129
M46I_I47V_I50V_L76V_V82F	-2.290375965
I50V_I54L_L76V_V82T_I84V	-2.291086332
I47V_I50V_I54M_L76V_V82T	-2.292557286
M46I_V82T_L90M	-2.293155454
V32I_M46I_I47V_I50V_I54L_V82T_L90M	-2.298493709
I50V_I54M_V82T_I84V	-2.299870024
V32I_I50V	-2.301135711
I54L_T74P_L76V_V82F_I84V	-2.301381204
V32I_I47V_I50V_I54M_I84V	-2.302771743
M46I_I47V_I50V_V82T_L90M	-2.304974296
I50V_I54M_L76V_V82F	-2.306160533
M46I_I47V_I54M_L76V_V82T_L90M	-2.308904589
I50V_I54L_L76V_I84V	-2.310403463
M46I_I50V_L76V_V82T	-2.31128038
V32I_I50V_I54L_V82F_L90M	-2.314734133
M46I_I50V_I54L_L76V_V82T_L90M	-2.314734133
I47V_I54L_L76V	-2.315991912
M46I_I47V_I50V_I54M_V82F_L90M	-2.316200925
I50V_L76V_V82F	-2.317473019
I50V_I54M_V82F_I84V	-2.317647413
I47V_I54M_L76V_V82T	-2.319141151
M46I_I47V_V82T	-2.319272509

I50V_L76V_V82T_I84V	-2.320159252
M46I_I47V_I50V_I54L_V82F_I84V	-2.32362034
L10F_V32I_I47V_T74P	-2.324279451
I47V_I50V_I54M_L76V	-2.325858128
V32I_I50V_I54L_L76V_V82T	-2.328715752
M46I_T74P_L76V	-2.32980854
M46I_I47V_I50V_I54M_V82F_I84V_L90M	-2.330649852
I47V_I50V_I54M_L76V_I84V_L90M	-2.332190182
M46I_I47V_I50V_I54L_L76V_V82T_I84V_L90M	-2.3326093
V82F	-2.333144788
M46I_I47V_I50V_I54L_L76V_V82F	-2.333911756
V32I_I54L_T74P	-2.334207993
I47V_I50V_I54M_L76V_V82T_I84V	-2.336818692
M46I_I50V_I54M_V82F_I84V	-2.337577767
M46I_I50V_V82F_I84V	-2.337957179
V32I_I54M_L76V_V82F_I84V	-2.338846389
I50V_I54M_L76V	-2.339112335
I54M_L76V_V82F_I84V	-2.341075762
V32I_I47V_T74P_I84V	-2.341811338
V32I_I50V_I54M_V82T_I84V	-2.341876499
V32I_M46I_I47V_I50V_V82T_I84V	-2.341987361
I54L_V82F_I84V	-2.344009083
M46I_I47V_I50V_L76V	-2.34639488
V32I_I84V	-2.347071087
V32I_M46I_I47V_T74P	-2.347452121
M46I_I47V_I50V_I54M_V82F	-2.348741097
M46I_I50V_L76V_V82F_I84V	-2.349634354
I47V_T74P_V82F_L90M	-2.349634354
V32I_M46I_I50V_I84V	-2.351223238
V32I_I50V_I54L_L76V_V82T_I84V	-2.352548481
M46I_I50V_I54M_L76V_I84V	-2.355129003
I47V_I54M_V82T	-2.35520235
V32I_M46I_I47V_I54L_I84V	-2.356771227
V32I_I50V_I54L_I84V	-2.357479022
V32I_M46I_I50V_I54M_V82F	-2.357712128
V32I_M46I_I47V_V82T	-2.359169697
I47V_T74P_V82T	-2.359179352
L10F_I47V_T74P_V82F	-2.361359345
V32I_I47V_I50V_I54M_L90M	-2.361855331
I47V_I50V_L76V_V82T	-2.361919566
V32I_I50V_L76V_V82F_L90M	-2.363219039
V32I_M46I_I50V_I54L_L76V_V82F_L90M	-2.363219039
M46I_I50V_I54M_V82T_I84V	-2.363291175

I54L_L76V	-2.364017665
I47V_I50V_I54L_T74P_V82F	-2.364845434
I50V_I54M_V82T	-2.365122999
V32I_I47V_I50V_I54M_L76V_V82T_I84V	-2.36547885
V32I_M46I_I54L_L76V_V82T	-2.365760643
V32I_M46I_I47V_I54M_L76V_I84V	-2.367345268
I54L_V82F_L90M	-2.368218798
V32I_L76V_V82T_I84V_L90M	-2.369091795
I54M_L76V_V82T	-2.369862785
V32I_I47V_I50V_I54L_L76V_L90M	-2.371491565
V32I_I47V_I50V_I54M_L76V_V82F	-2.373371675
V32I_M46I_I50V_L76V_I84V	-2.374609148
I50V_I54M_V82F_L90M	-2.375431973
M46I_I47V_V82T_I84V	-2.378567209
V32I_I47V_I54M_L90M	-2.379582294
I47V_I54L_T74P_L76V	-2.380097931
M46I_I54M_L76V_V82F	-2.381294113
M46I_I50V_V82T	-2.381780175
M46I_I50V_I54M_L76V_V82T_I84V	-2.382217344
I50V_I54L_I84V_L90M	-2.382247759
I47V_I50V_I54M_V82F_L90M	-2.383028397
I47V_I50V_I54M_V82T	-2.383360443
M46I_I47V_V82F	-2.383886663
V32I_I54M_L76V_V82T_I84V	-2.385899689
M46I_V82F_L90M	-2.387089079
V32I_M46I_I47V_I54M_V82T	-2.388727512
M46I_I47V_I50V_I54M_V82T_I84V_L90M	-2.390307757
M46I_I47V_I54M_V82T	-2.390537357
M46I_I47V_I54M_L90M	-2.390655471
V32I_I47V_I54M_L76V	-2.391283596
I47V_I54L_L76V_V82F	-2.392832715
V32I_M46I_I47V_I50V_I54L_L76V_L90M	-2.393915379
M46I_I47V_I50V_I54L_L76V_V82T	-2.394280488
V32I_M46I_I50V_V82T_L90M	-2.394280488
M46I_I54M_I84V_L90M	-2.394639216
M46I_I54M_L76V	-2.395792727
V32I_M46I_I47V_I50V_I54M_V82F_I84V	-2.396383573
M46I_I47V_I54L_I84V	-2.397339377
V32I_I47V_V82T	-2.398296166
V32I_I47V_I50V_I54M_V82T_I84V_L90M	-2.398296166
M46I_I50V_I54M_L76V	-2.398645254
V32I_M46I_I47V_L76V_V82T	-2.398853644
I50V_I54L_V82T_I84V	-2.401538141

M46I_I50V_I54L_V82F_I84V	-2.401661833
V32I_M46I_V82T	-2.4021392
V32I_I47V_I54M_V82T_I84V	-2.402518654
M46I_I50V_I54L_V82T_I84V	-2.402988154
I54M_V82T_I84V_L90M	-2.403275624
I54L_V82T_I84V	-2.404548498
V32I_I50V_I54M	-2.405229862
V32I_I47V_T74P_V82F	-2.405311639
V32I_M46I_I47V_I50V_I54L_V82T	-2.405498295
M46I_I47V_I54M_L76V	-2.405757239
V32I_M46I_I54L_V82T	-2.405815487
I47V_V82F	-2.406213832
V32I_I47V_I50V_I54L_T74P	-2.407493516
V32I_M46I_I50V_I54L_L76V_V82T	-2.407697082
M46I_I54L_V82T_L90M	-2.407814859
V32I_I50V_L76V_I84V	-2.409103819
V32I_M46I_L76V_V82T_I84V	-2.409641162
V32I_M46I_I47V_I50V_L76V_V82T_I84V_L90M	-2.410643689
I50V_I54L_L90M	-2.414098887
V32I_I47V_I50V_L76V_I84V_L90M	-2.415721154
M46I_I47V_I54L_L76V	-2.416415171
V32I_M46I_I50V_L76V_V82F	-2.416639034
M46I_I50V_I54M_I84V_L90M	-2.417396475
V32I_I50V_V82T_I84V_L90M	-2.417396475
V32I_M46I_I47V_I50V_I54M_L76V_I84V	-2.417667199
V32I_M46I_I47V_I50V_I54L_L76V	-2.418042487
V32I_M46I_I50V_I54M_L76V_V82F_I84V	-2.421667387
I47V_I54L_T74P_V82F_I84V	-2.421842846
M46I_L76V_V82F	-2.423097791
I54L_L76V_V82T_I84V	-2.424136079
V32I_M46I_I50V_I54M_V82T_I84V	-2.424164003
V32I_I47V_I54L_L76V	-2.424359354
V32I_M46I_I47V_I50V_I54L_V82T_I84V	-2.42443583
M46I_I47V_T74P_L76V	-2.425980383
V32I_M46I_I50V_V82T_I84V	-2.426176418
V32I_M46I_I47V_I50V_I54M_L90M	-2.428630303
V32I_M46I_I47V_L76V_I84V_L90M	-2.428677485
M46I_I47V_I50V_T74P	-2.430117374
V32I_M46I_I50V_L76V_V82T_I84V	-2.430611508
M46I_I47V_I50V_I54M_L76V	-2.431830587
V32I_M46I_I54M_V82T	-2.432561039
V32I_M46I_I47V_I50V_L76V_V82T_I84V	-2.432633882
M46I_I47V_I50V_I54M_V82T	-2.432791394

I47V_I50V_I54M_I84V	-2.433174885
V32I_I54M_L76V_V82T	-2.433265549
I54L_L76V_V82F_I84V	-2.433486688
I47V_I54M_V82F	-2.433995889
V32I_I47V_I50V_L76V_V82T_L90M	-2.433995889
V32I_M46I_I50V_I54L_V82T_L90M	-2.434250174
V32I_I54M_V82T	-2.434408493
I47V_I54M_T74P_V82F_I84V	-2.435752929
M46I_L76V_V82T_I84V_L90M	-2.4361092
V32I_M46I_I50V_I54L_L76V	-2.436156659
V32I_I47V_I54M_V82F_L90M	-2.436652377
V32I_M46I_I50V_I54L_L76V_V82F	-2.438209814
I47V_I50V_V82F_I84V	-2.438290002
M46I_V82F_I84V	-2.439448051
V32I_I47V_I50V_I54L_L76V	-2.439582498
M46I_I47V_L76V_I84V_L90M	-2.440077483
I47V_I54L_L76V_V82T_I84V	-2.440338393
M46I_L76V_I84V	-2.44102732
V32I_M46I_I47V_V82T_I84V_L90M	-2.441127064
V32I_M46I_I47V_I50V_I54M_V82T_L90M	-2.441870157
V32I_I47V_I54L_V82T	-2.442752089
M46I_I47V_L76V	-2.443753615
I54M_L76V_V82F	-2.443897669
I47V_I54L_L76V_I84V	-2.443946569
V32I_M46I_I50V_L76V	-2.445344639
I47V_I50V_I54M_V82T_I84V_L90M	-2.445344639
L76V_L90M	-2.447110573
I47V_I54L_V82F_I84V	-2.447591062
M46I_I50V_I54M_I84V	-2.448362347
I54M_T74P	-2.449231785
M46I_I50V_I54M_V82T_I84V_L90M	-2.449888743
V32I_I50V_L76V_V82F_I84V_L90M	-2.449900961
M46I_I47V_I54L_L76V_V82T	-2.450362888
V32I_M46I_I54L_V82F	-2.451773014
V32I_M46I_L76V_I84V	-2.451926312
V32I_I47V_I50V_L76V	-2.453911178
M46I_I47V_I50V_V82T_I84V	-2.454010592
M46I_I50V_I54M_V82F	-2.454295754
I47V_I50V_I54M_V82F	-2.45434637
M46I_I50V_L76V_V82T_I84V	-2.454376152
V32I_I47V_I54L_I84V	-2.456190988
I47V_I54M_L76V	-2.456577033
V32I_M46I_I50V_I54M_I84V	-2.45702118

I47V_I54M_L76V_V82F_I84V	-2.457600735
I47V_I50V_V82T	-2.458067729
I47V_I54M_L76V_V82F	-2.459263944
M46I_I54L_V82T_I84V_L90M	-2.460129052
I47V_I54M_I84V	-2.462173637
I47V_I50V_I84V	-2.462821124
I47V_T74P_L76V	-2.463201117
V32I_I47V_I50V_I54L_L76V_V82F	-2.464186587
I47V_I54M_L90M	-2.464289912
V32I_M46I_I50V_V82T_I84V_L90M	-2.464289912
V32I_M46I_I47V_I54L_L76V	-2.467518058
I50V_L76V	-2.468129921
V32I_M46I_I54M_L76V_V82T_I84V	-2.468243328
I47V_L76V_V82T	-2.468284684
I47V_I50V_L76V	-2.468322756
V32I_T74P	-2.468495855
M46I_I50V_V82F_I84V_L90M	-2.469134616
I50V_I54L_L76V	-2.469575914
I50V_I54L_V82F_I84V	-2.470235279
V32I_I50V_I54M_V82F_L90M	-2.470235279
M46I_I50V_I54L_V82T_I84V_L90M	-2.470293388
M46I_I54L_V82F_I84V	-2.470435615
V32I_L76V_V82F_L90M	-2.471385497
I54L_T74P_L76V	-2.472321481
M46I_I54M_L76V_V82T	-2.472938816
I47V_I50V_I54M_V82T_I84V	-2.474363332
I50V_I54L_V82F	-2.47453636
M46I_I47V_I54L_L76V_V82F_I84V	-2.475027274
M46I_I47V_I50V_I54M_L76V_V82F_I84V_L90M	-2.47688319
I50V_I54M_I84V_L90M	-2.476956936
V32I_M46I_I47V_I54L_V82T_I84V	-2.477140119
V32I_M46I_I54M_V82T_I84V	-2.4775001
V32I_L76V_I84V	-2.478646235
I47V_T74P_V82F	-2.479433262
V32I_I47V_I54M_L76V_V82F	-2.479733117
M46I_I54L_L76V_I84V_L90M	-2.480395081
I50V_I54L_L76V_I84V_L90M	-2.481065555
M46I_I54M_L76V_V82T_I84V	-2.481343227
M46I_I47V_L76V_I84V	-2.482412078
M46I_I50V_L76V_V82F_L90M	-2.483135601
V32I_M46I_L76V_V82F_I84V	-2.483239809
V32I_M46I_I47V_I50V_I54M_L76V_V82T_I84V	-2.48356517
M46I_I47V_I50V_I54L_L76V	-2.483574992

I47V_V82F_I84V	-2.48367921
I47V_L76V	-2.484188188
I54M_L76V_V82T_I84V	-2.484987464
V32I_I47V_I50V_L76V_V82T	-2.485003877
V32I_M46I_I47V_I50V_I54L_L90M	-2.485275
V32I_M46I_I47V_I54M_L76V_V82F_L90M	-2.485275
V32I_I47V_V82T_I84V	-2.486077956
I50V_I54M_L76V_I84V	-2.486266218
V32I_I47V_I50V_I54L_V82F	-2.48727376
V32I_M46I_I47V_I50V_L76V	-2.488230508
I47V_T74P	-2.489969406
M46I_I47V_I50V_I54L_L76V_V82T_I84V	-2.490374158
V32I_M46I_I47V_I50V_I54L_L76V_V82T	-2.490879111
I47V_I54L_V82F	-2.49101853
M46I_I50V_I54M_V82T	-2.491070322
M46I_I47V_I50V_V82T_I84V_L90M	-2.491569564
V32I_I47V_I50V_I54L_L76V_V82T_L90M	-2.491569564
M46I_I47V_I50V_I54M_L76V_V82F_L90M	-2.491569564
M46I_I54L_L76V_V82T_L90M	-2.491615516
I47V_I50V_I54L_L76V_V82T_I84V	-2.491712436
V32I_I47V_I54M_L76V_I84V	-2.491903495
V32I_I47V_I50V_I54L_V82F_L90M	-2.491903495
M46I_I47V_I54M_L76V_L90M	-2.492051578
V32I_M46I_L76V	-2.493082809
V32I_T74P_V82T	-2.493082809
M46I_T74P_V82F_I84V	-2.493217748
M46I_I50V_I54L_L90M	-2.494376896
V32I_M46I_I50V_I54L_V82T	-2.494637077
V32I_I47V_I50V_V82T_I84V	-2.495639742
M46I_I47V_I50V_I54L_V82T	-2.4964324
V32I_M46I_I47V_I50V_V82F	-2.496780727
V32I_I50V_L76V_V82F_I84V	-2.496816974
M46I_I47V_I50V_I54L_L76V_I84V	-2.49714855
I47V_I50V_I54L_L76V_V82T	-2.497468813
M46I_I47V_I54L_V82T	-2.497898144
M46I_I50V_I54M_L76V_V82F_I84V_L90M	-2.497898144
I54L_L76V_L90M	-2.498534066
I47V_L76V_V82F_I84V	-2.499142988
V32I_I47V_V82T_I84V_L90M	-2.499360063
V32I_M46I_I50V_I54M_L76V_V82T_I84V	-2.49938389
I47V_I50V_I54M_V82F_I84V	-2.499774127
V32I_I50V_L76V_V82T_I84V	-2.500351209
V32I_M46I_I47V_I50V_I54M_L76V_V82T	-2.50037071

V32I_T74P_V82F_I84V	-2.50037071
M46I_I47V_I50V_V82F	-2.501301221
M46I_I54M_V82T_I84V_L90M	-2.501565948
M46I_I47V_I50V_L76V_V82F_I84V	-2.501908649
V32I_M46I_I50V_L76V_V82F_I84V	-2.502526891
M46I_I47V_I50V_I54L_L76V_V82F_I84V_L90M	-2.503070519
V32I_M46I_L76V_V82T_I84V_L90M	-2.503085497
M46I_I47V_I50V_I54L_L76V_V82F_I84V	-2.503900422
V32I_M46I_I47V_I50V_I84V_L90M	-2.503900422
V32I_I50V_I54M_L76V_I84V	-2.504090893
V32I_M46I_V82T_I84V_L90M	-2.504548192
V32I_I47V_L76V	-2.504571884
M46I_I47V_I54M_L76V_I84V	-2.505166068
V32I_M46I_I50V_V82F_L90M	-2.505166068
V32I_M46I_I47V_I50V_L76V_I84V_L90M	-2.505166068
I50V_I54L_V82T	-2.505981621
V32I_I54L_V82F_I84V	-2.506277322
V32I_M46I_I54L_L76V	-2.50633531
M46I_I47V_I50V_I54M_L90M	-2.508409062
V32I_M46I_I50V_I54L_L76V_V82F_I84V	-2.509371631
V32I_M46I_I47V_I50V_I54M_L76V_L90M	-2.509710736
V32I_I47V_L76V_I84V	-2.509770305
M46I_L76V_V82F_I84V	-2.510016784
V32I_M46I_L76V_V82F	-2.51013954
M46I_I47V_I54L_L76V_V82T_I84V	-2.51023767
V32I_I47V_I50V_I54M_V82F_L90M	-2.510693508
M46I_V82F_I84V_L90M	-2.511164325
V32I_I54L_T74P_L76V	-2.511164325
M46I_I47V_I54L_V82F	-2.511958694
V32I_M46I_I50V_I54M_L76V_L90M	-2.512453831
M46I_I47V_I54M_V82T_I84V_L90M	-2.512779968
M46I_I47V_I50V_I54L_V82F	-2.513037267
V32I_M46I_I47V_I50V_I54M_L76V_V82F	-2.513079076
V32I_M46I_I50V_V82T	-2.513347747
M46I_I47V_I50V_L76V_L90M	-2.51358096
V32I_I47V_I50V_V82T_I84V_L90M	-2.51446091
V32I_M46I_I54L_V82F_I84V	-2.515018753
M46I_I50V_I54M_L76V_V82F_L90M	-2.515067845
V32I_M46I_I47V_I54M_I84V	-2.515293378
V32I_M46I_I54L_L76V_V82F_I84V	-2.515929494
I50V_I84V	-2.516933342
V32I_I47V_V82F_I84V	-2.517646403
V32I_I50V_I54M_L76V	-2.51821977

V32I_M46I_I50V_I54M_L90M	-2.519361058
M46I_I47V_V82T_L90M	-2.519941494
V32I_I54M_L76V_V82T_I84V_L90M	-2.520342459
I47V_I50V_I54L_V82T	-2.520981104
I50V_I54L_L76V_V82F	-2.521046894
V32I_I54L_V82T_I84V	-2.521976939
V32I_M46I_V82F_I84V_L90M	-2.522111076
V32I_T74P_I84V	-2.522276959
V32I_M46I_I47V_I50V_I54L_L76V_V82T_L90M	-2.522726224
M46I_I47V_I54M_V82F_I84V	-2.524350421
M46I_I47V_I54L_V82T_I84V	-2.524571493
I50V_I54M_I84V	-2.524601451
I54M_L76V_V82T_I84V_L90M	-2.524751218
I47V_I54M_T74P_V82F	-2.524865623
I50V_I54M_L76V_V82T_I84V	-2.525055559
V32I_M46I_I50V_L76V_L90M	-2.526515149
M46I_I50V_V82F	-2.527083587
V32I_M46I_I47V_V82T_I84V	-2.528482036
V32I_M46I_I50V_I54L_V82F_I84V_L90M	-2.529545702
M46I_I50V_V82T_I84V	-2.529815617
M46I_I47V_I50V_I54M_V82T_L90M	-2.530565469
V32I_I47V_I54M_T74P	-2.531550318
T74P_V82F	-2.53175533
I47V_V82T_I84V	-2.532147614
V32I_I47V_I54M_V82F	-2.533894834
V32I_M46I_V82F_I84V	-2.535958288
V32I_I47V_I50V_I54L_V82F_I84V	-2.536375436
I47V_I50V_I54L_L76V_I84V_L90M	-2.536375436
V32I_I47V_I50V_I54M_T74P_V82F_I84V	-2.536582883
M46I_I47V_I50V_I54M_L76V_V82T_I84V_L90M	-2.536582883
V32I_M46I_I54M_T74P	-2.536582883
V32I_M46I_I47V_I50V_V82T_I84V_L90M	-2.537467351
V32I_I50V_I54M_L76V_V82F_L90M	-2.537872339
V32I_M46I_I47V_V82F	-2.538541287
V32I_I50V_I54L_L76V_V82T_L90M	-2.53905732
V32I_I47V_I50V_I54L_L76V_I84V	-2.540107277
V32I_I47V_I50V_I54M_V82T	-2.540917195
V32I_M46I_I47V_I54L_L76V_V82F	-2.54132064
V32I_I47V_I50V_I54M_V82T_L90M	-2.541700118
V32I_I47V_L76V_V82T_I84V	-2.542396775
M46I_I50V_I84V	-2.54270774
I47V_I50V_V82T_I84V	-2.542907453
I54M_L76V_V82T_L90M	-2.543227027

V32I_I50V_I54L_L76V_V82F_L90M	-2.543416963
I47V_I54L_I84V	-2.544023814
I54L_L76V_V82T	-2.544095702
I47V_I50V_V82F	-2.545664311
V32I_I54M_L76V_I84V	-2.545788532
M46I_I47V_I54M_L76V_V82T	-2.546612834
V32I_I47V_I54L_V82F_I84V	-2.546675985
V32I_I47V_T74P	-2.547262752
V32I_I47V_I54L_L76V_I84V_L90M	-2.547744368
M46I_I47V_I50V_L76V_I84V	-2.547781945
V32I_M46I_I50V_L76V_V82F_I84V_L90M	-2.548488206
I54L_V82T_I84V_L90M	-2.549159013
V32I_M46I_I54L_I84V	-2.550242846
I50V_I54L_L76V_V82T	-2.550644458
M46I_I47V_L76V_V82F	-2.550863774
M46I_I47V_I50V_I54M_L76V_V82F_I84V	-2.550902769
M46I_I54L_T74P_V82F_I84V	-2.550914457
M46I_I47V_I54M_L76V_V82F_I84V_L90M	-2.551333661
V32I_I47V_I50V_L76V_V82T_I84V	-2.551729174
M46I_I50V_I54L_L76V_I84V	-2.552074811
M46I_L76V_V82F_I84V_L90M	-2.552239168
V32I_M46I_I47V_I50V_I54M_L76V	-2.552378487
V32I_M46I_I47V_I54L_V82F	-2.553154027
V32I_I50V_I54L_V82T_I84V_L90M	-2.553616222
V32I_M46I_I47V_I50V_L76V_I84V	-2.554054132
I50V_L76V_V82T_I84V_L90M	-2.555700714
V32I_M46I_I47V_I50V_L76V_V82T_L90M	-2.555700714
I50V_V82F	-2.555921098
V32I_M46I_I54L_L76V_V82T_I84V	-2.558152802
I54L_T74P_V82F_I84V	-2.558339018
M46I_I47V_I50V_I54L_L90M	-2.558541675
I47V_I50V_L90M	-2.559058037
V32I_M46I_I54M_I84V	-2.559873994
M46I_I50V_L90M	-2.559943599
V32I_I47V_I50V_I54L_L76V_V82T_I84V	-2.560124294
V32I_I47V_I54M_V82T	-2.560232931
M46I_I54L_L76V_V82F	-2.560729122
M46I_I50V_I54M_L76V_V82F	-2.560730195
V32I_T74P_L76V	-2.561029548
V32I_I54L_L76V	-2.561754194
V32I_I47V_I54M_V82T_I84V_L90M	-2.561958222
M46I_I54L_L76V_V82T	-2.561992653
V32I_I50V_I84V	-2.562062136

M46I_I47V_I54L_L76V_V82F	-2.562731435
I47V_I50V_I54L_V82F	-2.562908881
M46I_I54M_T74P_I84V	-2.562920011
M46I_I47V_I54M_I84V	-2.564810282
V32I_M46I_I54M_L90M	-2.564862782
M46I_I47V_I54M_L76V_V82F	-2.565337896
V32I_M46I_I47V_I54L_L76V_I84V	-2.566624668
M46I_I47V_I50V_I54L_I84V	-2.567569896
V32I_M46I_I47V_V82F_I84V	-2.568816942
V32I_I47V_L76V_V82F	-2.569088371
M46I_I54L_L76V_V82T_I84V_L90M	-2.569458506
V32I_M46I_I47V_I54L_V82T	-2.570851678
V32I_M46I_I47V_I54L_L76V_V82T_I84V_L90M	-2.571494981
M46I_I47V_I50V_I54M_V82F_I84V	-2.57156697
M46I_I50V_I54M_L76V_V82T	-2.572623563
I47V_L76V_V82F	-2.573695951
V32I_M46I_I54L_L76V_I84V_L90M	-2.573695951
V32I_M46I_I47V_I54M_L76V	-2.575096816
M46I_I50V_I54L_L76V_V82T_I84V	-2.576375173
M46I_I47V_I50V_L76V_V82T	-2.576673135
V32I_M46I_I47V_V82F_I84V_L90M	-2.577202883
I50V_V82T	-2.577478785
V32I_M46I_I50V_I54M_L76V_V82F_I84V_L90M	-2.578203872
I47V_I50V_I54M_L90M	-2.578334406
V32I_M46I_I54M_L76V_V82F_I84V_L90M	-2.578388686
V32I_V82F	-2.578540291
I50V_I84V_L90M	-2.578647943
V32I_M46I_I47V_I50V_I54M_V82F	-2.578985284
I47V_I54M_V82F_I84V	-2.57956529
V32I_I47V_I50V_I54M_V82F	-2.579648859
M46I_I47V_V82F_I84V	-2.579687311
V32I_I47V_I50V_I54L_V82T_I84V	-2.580929287
V32I_I50V_I54M_I84V	-2.581432937
V32I_M46I_I47V_I50V_V82T	-2.58144901
M46I_I50V_L76V_V82F_I84V_L90M	-2.58150946
I47V_I54M_L76V_I84V	-2.584939622
V32I_M46I_I54M_L76V_I84V	-2.584939911
V32I_M46I_V82T_L90M	-2.584939911
I50V_I54M_L76V_V82F_I84V	-2.585551306
V32I_M46I_I47V_I54M_L90M	-2.585565749
V32I_M46I_I50V_I54M_L76V_I84V	-2.585637501
V32I_I47V_I54M_I84V	-2.586015417
I47V_I54M_L76V_V82F_L90M	-2.586855461

V32I_I54L_V82F	-2.58728653
V32I_I54M_V82T_I84V	-2.588110664
V32I_M46I_I50V_I54M_L76V_V82T	-2.58924895
V32I_I47V_I50V_I54M_I84V_L90M	-2.589716886
I54L_L76V_I84V_L90M	-2.589966563
M46I_I47V_I50V_I54L_L76V_I84V_L90M	-2.591698367
V32I_I47V_I50V_L76V_L90M	-2.592783447
M46I_I54M_L76V_L90M	-2.592949907
M46I_I54L_I84V_L90M	-2.593273928
I47V_I50V_L76V_V82F_I84V_L90M	-2.593487734
I54M_L76V_L90M	-2.593667849
V32I_M46I_I47V_I50V_L76V_V82F_I84V	-2.593770514
V32I_M46I_I54M_L76V	-2.594195371
V32I_L76V_V82T	-2.594308645
V32I_M46I_I47V_I54M_V82T_I84V	-2.594323416
I47V_L76V_I84V_L90M	-2.594488123
V32I_I47V_I50V_I84V	-2.594511654
M46I_I50V_I54L_L76V_V82F_L90M	-2.594775453
M46I_I54M_L76V_V82F_I84V	-2.595742618
V32I_I50V_I54L_L90M	-2.597472969
V32I_I47V_I54M_L76V_V82T	-2.597949069
V32I_I47V_I54L_V82F	-2.597992565
V32I_M46I_I54M_V82F	-2.598509756
V32I_I54M_L76V_V82F_I84V_L90M	-2.599097544
V32I_M46I_I50V_I54L_V82T_I84V	-2.599848376
M46I_I47V_I50V_V82F_I84V	-2.600472247
V32I_M46I_I54L_L76V_I84V	-2.600502957
M46I_I50V_V82T_L90M	-2.600667111
M46I_I50V_L76V	-2.600932822
I54M_I84V_L90M	-2.601304997
V32I_M46I_I47V_I50V_L76V_V82F_L90M	-2.601458205
I50V_I54M_L76V_I84V_L90M	-2.601838077
V32I_I54L_I84V	-2.601991912
V32I_I47V_I50V_I54L_I84V	-2.603350649
V32I_M46I_I54M_L76V_V82F	-2.603354499
V32I_M46I_I47V_I50V_I54L_L76V_V82F	-2.60384872
I47V_I50V_I54L_I84V	-2.604192761
V32I_M46I_I54L_V82T_I84V	-2.605085757
M46I_I50V_L76V_I84V	-2.605369717
M46I_I47V_I54L_V82F_I84V	-2.605998826
V32I_I47V_I54M_L76V_V82T_L90M	-2.606920388
V32I_I50V_I54L_V82F	-2.607117819
V32I_M46I_I50V_L76V_V82F_L90M	-2.607643917

V32I_M46I_I47V_I50V_L76V_V82F	-2.607725659
V32I_L76V_V82T_I84V	-2.609018847
I54L_L76V_I84V	-2.61008568
V32I_M46I_I54L_L90M	-2.6103164
I47V_I54L_L76V_V82F_I84V	-2.610681957
M46I_I50V_I54L_I84V	-2.610884049
M46I_I47V_I50V_I54L_L76V_L90M	-2.611003522
I47V_L76V_I84V	-2.611027661
M46I_I54M_V82F_L90M	-2.611632997
I54L_L76V_V82T_L90M	-2.612022396
V32I_I47V_I50V_L76V_V82F	-2.612410381
V32I_M46I_I47V_I50V_I54M_L76V_V82F_I84V	-2.614325898
I47V_I54M_I84V_L90M	-2.614346786
V32I_I50V_I54M_L76V_V82F	-2.615389673
I47V_I50V_I54M_L76V_V82T_I84V_L90M	-2.615764129
I50V_I54M_L76V_V82F_I84V_L90M	-2.615764129
I47V_I54M_T74P	-2.616357414
M46I_L76V_V82T	-2.616539032
V32I_I47V_I54M_L76V_L90M	-2.617127836
I50V_I54L_V82T_L90M	-2.618464131
V32I_I47V_I50V_I54M_L76V_L90M	-2.618975909
V32I_I47V_I84V_L90M	-2.619231963
M46I_I54L_V82T_I84V	-2.619655634
V32I_I47V_I54L_V82T_I84V	-2.620097067
I47V_I50V_I54M_I84V_L90M	-2.620343549
M46I_I47V_L76V_V82F_I84V	-2.620589621
M46I_I50V_I54L_V82T	-2.621374158
V32I_M46I_T74P_V82F_I84V	-2.621945881
I50V_I54L_L76V_L90M	-2.623039824
I47V_I54L_T74P_V82F	-2.623202882
M46I_I47V_L76V_V82T	-2.623377956
V32I_M46I_I54M_L76V_V82F_I84V	-2.624002982
V32I_I47V_I54L_L76V_V82F_I84V	-2.624086136
V32I_V82F_I84V	-2.624969429
I54M_L76V_I84V	-2.624993809
M46I_I47V_I50V_L76V_V82T_L90M	-2.624993809
M46I_I50V_I54L_L76V_V82T	-2.626931847
M46I_I50V_I54M_V82F_L90M	-2.62695518
I54M_V82F_I84V_L90M	-2.627834184
V32I_M46I_L76V_V82F_I84V_L90M	-2.628545794
I47V_I50V_L76V_I84V	-2.628575079
V32I_M46I_I54M_V82T_L90M	-2.62857514
V32I_I47V_I50V_I54L_L90M	-2.628840975

M46I_I54M_L76V_V82T_I84V_L90M	-2.62949394
V32I_M46I_I54L_V82T_L90M	-2.62967277
I50V_I54M_V82T_L90M	-2.630004568
V32I_I47V_I50V_V82T_L90M	-2.630004568
I47V_I50V_I54M_L76V_V82F_I84V	-2.630047759
M46I_I47V_I50V_I54M_L76V_I84V_L90M	-2.630644114
V32I_I54L_L90M	-2.630791626
V32I_I47V_I50V_I54L_V82T	-2.631382519
I47V_I50V_L76V_V82T_I84V	-2.632512072
V32I_M46I_I50V_I54M_V82T	-2.634460432
V32I_M46I_I47V_V82T_L90M	-2.634604046
V32I_L76V_V82F	-2.635042302
M46I_I47V_L76V_V82T_I84V	-2.635177001
V32I_V82T	-2.636063803
V32I_L76V	-2.6362339
M46I_I47V_I54L_L76V_V82F_I84V_L90M	-2.63709181
V32I_I50V_L76V_V82T_L90M	-2.638441771
V32I_M46I_I47V_T74P_L76V_L90M	-2.638441771
V32I_I50V_I54L_L76V_I84V	-2.638517767
I50V_I54M_L76V_L90M	-2.640778706
M46I_I54M_V82F_I84V	-2.640978456
M46I_I47V_T74P_V82F	-2.642384662
V32I_M46I_I47V_I54M_V82F_I84V_L90M	-2.642589502
V32I_M46I_I50V_I54L_L76V_V82T_L90M	-2.643792852
V32I_I47V_I50V_L76V_V82F_I84V_L90M	-2.643792852
V32I_M46I_I54L_L76V_V82F	-2.644499131
M46I_I47V_I50V_I54M_L76V_V82T	-2.644658476
I47V_I54M_L76V_V82F_I84V_L90M	-2.64675829
M46I_I47V_T74P_V82T	-2.64685722
V32I_I50V_I54M_L76V_V82F_I84V	-2.648157105
M46I_I47V_T74P_I84V	-2.64820179
I47V_I54M_L76V_I84V_L90M	-2.648737295
V32I_M46I_I54L_V82F_I84V_L90M	-2.649760003
V32I_I50V_V82F	-2.649763308
I47V_T74P_L76V_V82F	-2.649778768
I50V_I54M_V82T_I84V_L90M	-2.649981695
M46I_I54L_L76V_V82F_I84V	-2.650820475
I50V_I54M_L76V_V82T	-2.651131791
I50V_L76V_V82T	-2.651276993
I47V_I50V_I54L_L76V_V82F_I84V_L90M	-2.651537069
M46I_I47V_I50V_I54M_L76V_L90M	-2.65205461
V32I_M46I_I47V_I50V_V82T_L90M	-2.652839895
V32I_M46I_I50V_L76V_V82T_L90M	-2.652974449

V32I_M46I_I47V_L76V_V82T_I84V_L90M	-2.652995727
V32I_I47V_I54L_L76V_V82F	-2.653076428
V32I_M46I_I47V_I50V_V82F_I84V	-2.653130477
V32I_I47V_I54M_L76V_V82T_I84V	-2.654542626
V32I_I47V_I54L_L76V_V82T_I84V	-2.655130276
M46I_I54M_V82F_I84V_L90M	-2.655636142
V32I_I47V_I50V_I54M_L76V_V82T_I84V_L90M	-2.655815867
V32I_I50V_I54M_L76V_L90M	-2.657156814
V32I_I47V_I54M_L76V_I84V_L90M	-2.657156814
I50V_I54M_L76V_V82F_L90M	-2.658477058
V32I_V82T_I84V	-2.659069142
V32I_M46I_I47V_I50V_I54L_V82F_L90M	-2.66020484
V32I_I47V_I50V_V82F_I84V	-2.660866796
V32I_I50V_I54L_L76V_V82F_I84V	-2.661181817
V32I_M46I_I47V_I54L_L76V_V82F_I84V	-2.661990219
M46I_I47V_I50V_L76V_V82F_L90M	-2.661990219
V32I_M46I_I47V_I54M_L76V_V82F_I84V	-2.662094599
V32I_M46I_I54M_L76V_I84V_L90M	-2.662780904
M46I_I47V_I50V_I54L_L76V_V82T_L90M	-2.662885327
V32I_I54L_L76V_V82F	-2.663502126
V32I_I47V_I50V_I54L_I84V_L90M	-2.664249034
V32I_M46I_I47V_I50V_I54L_I84V	-2.664615593
V32I_I50V_I54M_V82F	-2.664646706
M46I_L76V_V82T_I84V	-2.665353707
M46I_I50V_I54L_L76V_V82T_I84V_L90M	-2.665891949
M46I_I54L_T74P_L76V_V82F	-2.666693573
V32I_I50V_I54L_V82T_L90M	-2.667239858
M46I_I54M_V82T_L90M	-2.668579588
V32I_I54L_L76V_V82T_I84V	-2.669362071
I50V_I54L_L76V_V82F_I84V	-2.669723226
V32I_M46I_I50V_I54L_L76V_L90M	-2.670121791
M46I_I50V_I54L_L76V_I84V_L90M	-2.670121791
V32I_T74P_V82F	-2.670867523
M46I_I47V_I50V_I84V	-2.67104038
I47V_I50V_I54L_V82T_I84V_L90M	-2.671682886
M46I_L76V_V82T_L90M	-2.67252156
I47V_I50V_I54M_V82T_L90M	-2.673081906
M46I_I47V_L76V_V82F_I84V_L90M	-2.673161579
I47V_I54L_L76V_V82T	-2.673454261
V32I_M46I_I47V_I50V_I54L_L76V_V82F_I84V_L90M	-2.673527178
V32I_I50V_I54M_V82F_I84V	-2.674956205
V32I_M46I_I54M_V82T_I84V_L90M	-2.675025515
V32I_M46I_I54M_I84V_L90M	-2.675818129

V32I_M46I_I47V_I54M_L76V_V82F	-2.677013213
V32I_M46I_V82F	-2.677585769
V32I_M46I_I50V_I54M_L76V	-2.677624401
M46I_I54L_T74P_V82F	-2.677733268
M46I_I50V_L76V_I84V_L90M	-2.677733268
I50V_L76V_I84V_L90M	-2.678200755
V32I_I50V_V82F_I84V	-2.67861091
I47V_I54M_V82F_L90M	-2.678896996
I47V_I50V_I54L_V82F_I84V	-2.679131341
V32I_M46I_I47V_L76V_V82T_I84V	-2.679647654
I54M_V82F_L90M	-2.680483093
I50V_V82T_I84V_L90M	-2.680639451
L10F_V32I_I47V_T74P_V82F	-2.680639451
I47V_I50V_L76V_V82F_I84V	-2.680897435
V32I_I54L_L76V_I84V_L90M	-2.681215127
I47V_I50V_I54M_L76V_V82F_L90M	-2.682710918
M46I_I47V_I54M_V82T_I84V	-2.683709442
V32I_M46I_I50V_I54L_V82F	-2.684415249
V32I_M46I_I54M_L76V_V82T	-2.684936502
V32I_M46I_I47V_I54L_V82F_I84V	-2.685985252
V32I_I50V_L76V	-2.685986618
M46I_I47V_V82F_I84V_L90M	-2.687307834
I50V_I54L_V82F_I84V_L90M	-2.687723739
I50V_I54M_L90M	-2.688034269
V32I_I47V_I50V_I54L_L76V_I84V_L90M	-2.691113787
V32I_M46I_I47V_T74P_V82F_I84V	-2.691238488
V32I_M46I_I47V_I50V_I54M_I84V	-2.691776878
M46I_T74P_V82F	-2.692269178
V32I_M46I_I47V_L76V	-2.694063693
V32I_M46I_I47V_I50V_I54M_V82F_I84V_L90M	-2.694945375
M46I_I47V_I54L_I84V_L90M	-2.695011869
M46I_I47V_I54M_L76V_V82T_I84V	-2.696191118
V32I_M46I_I47V_I54M_L76V_I84V_L90M	-2.696433718
M46I_I50V_I54L_V82F_I84V_L90M	-2.696905886
I50V_V82T_I84V	-2.697015991
V32I_M46I_I47V_I50V_I54L_V82F_I84V	-2.699250843
V32I_M46I_I47V_I54M_V82F_L90M	-2.699250843
I47V_I54M_L76V_V82T_I84V	-2.699844664
M46I_I47V_I50V_I54L_V82T_L90M	-2.700799129
V32I_M46I_I47V_I50V_I54L_L76V_V82T_I84V	-2.701104416
V32I_M46I_I50V_I54L_V82T_I84V_L90M	-2.701222709
I47V_I50V_I54L_L76V_I84V	-2.701394554
V32I_I47V_I54L_L76V_L90M	-2.701622558

I54M_V82T_L90M	-2.702677725
V32I_I54M_V82F	-2.703284974
I50V_L90M	-2.70411396
V32I_M46I_I47V_L76V_I84V	-2.705156736
V32I_M46I_I47V_I54M_L76V_V82T	-2.705713049
M46I_I47V_I50V_L76V_V82F_I84V_L90M	-2.706844598
I50V_L76V_V82F_L90M	-2.707628564
V32I_M46I_I50V_L90M	-2.707628564
I47V_I50V_I54M_L76V_L90M	-2.707913535
M46I_I47V_I54M_T74P	-2.708549675
M46I_I54M_V82T_I84V	-2.71009627
V32I_I47V_I50V_V82F	-2.710872025
V32I_I50V_I54M_V82T_I84V_L90M	-2.711673684
V32I_M46I_I47V_V82F_L90M	-2.714037849
V32I_I47V_I50V_I54M_L76V_V82F_L90M	-2.714037849
V32I_M46I_I47V_I50V_V82F_I84V_L90M	-2.71426045
V32I_I47V_I50V_I54M_L76V	-2.714655623
V32I_M46I_I50V_I54M_V82T_L90M	-2.715148579
M46I_I47V_I54L_V82T_I84V_L90M	-2.715148579
V32I_M46I_I50V_I54M_V82F_L90M	-2.718426471
V32I_M46I_I54L_I84V_L90M	-2.718426471
V32I_M46I_I47V_I54L_L76V_V82T_I84V	-2.719692735
I47V_I50V_I54L_L76V_V82F_I84V	-2.720839023
V32I_M46I_I54L_L76V_V82F_I84V_L90M	-2.722633819
V32I_I47V_I50V_I54M_L76V_I84V	-2.724314629
V32I_I47V_L76V_V82T	-2.72492704
I50V_I54L_L76V_V82F_I84V_L90M	-2.72632472
M46I_I47V_I50V_I54L_V82T_I84V	-2.727122243
V32I_M46I_I47V_I54L_L76V_V82T	-2.728023137
I54L_V82T_L90M	-2.72868722
V32I_M46I_I47V_I50V_I54M_V82T	-2.729319329
M46I_I50V_I54M_L90M	-2.730001065
V32I_I54L_L76V_V82F_L90M	-2.730482105
V32I_I50V_L90M	-2.730635131
I54L_I84V_L90M	-2.731050114
V32I_M46I_I47V_I50V_L90M	-2.731550208
V32I_M46I_I47V_L76V_V82T_L90M	-2.732572608
V32I_M46I_I47V_I54M_V82F	-2.733304858
V32I_I54L_V82T	-2.733621636
M46I_I47V_I50V_I54M_I84V_L90M	-2.733821168
I54L_L76V_V82F_L90M	-2.734834543
V32I_I54M_L76V	-2.734972314
M46I_I47V_I50V_I54L_I84V_L90M	-2.735172431

V32I_I47V_L76V_V82F_L90M	-2.735776461
M46I_I47V_I50V_I54M_L76V_I84V	-2.737985537
V32I_I47V_I50V_L90M	-2.738115938
V32I_I50V_L76V_I84V_L90M	-2.740670123
M46I_I50V_L76V_L90M	-2.740670123
V32I_I47V_I50V_I54M_L76V_V82F_I84V	-2.740845171
V32I_I54L_L76V_L90M	-2.741020078
M46I_I50V_I54L_L76V_V82F	-2.741323572
M46I_I54M_L76V_I84V	-2.741585699
V32I_I47V_I54M_V82T_L90M	-2.741734038
V32I_I50V_I54L_L76V	-2.741797113
V32I_M46I_I47V_I50V_L76V_V82T	-2.743367639
V32I_I47V_I54L_L76V_V82T_I84V_L90M	-2.743367639
V32I_M46I_I54M_V82F_I84V	-2.744052578
I47V_L76V_V82F_I84V_L90M	-2.745561716
I50V_I54L_I84V	-2.746061547
I47V_I54L_V82F_L90M	-2.746669354
V32I_M46I_I50V_V82F	-2.747051945
V32I_M46I_I47V_I54M_L76V_L90M	-2.74728322
I50V_I54L_L76V_V82T_I84V_L90M	-2.74758624
V32I_I54L_L76V_V82T	-2.747772055
V32I_M46I_I47V_I54L_L76V_V82T_L90M	-2.747967551
I47V_I54M_L76V_V82T_L90M	-2.748377044
V32I_I50V_V82F_I84V_L90M	-2.749577842
I47V_I54L_I84V_L90M	-2.749845868
M46I_I47V_T74P	-2.749998505
I50V_L76V_V82F_I84V	-2.750276147
M46I_I54L_T74P_L76V_I84V	-2.750276147
V32I_I50V_I54M_V82T_L90M	-2.750680257
V32I_M46I_I47V_I50V_I54L_V82F_I84V_L90M	-2.751019416
V32I_M46I_I54L_V82T_I84V_L90M	-2.751223073
I47V_L76V_V82F_L90M	-2.751355321
V32I_M46I_I47V_I54L_V82T_L90M	-2.752905915
I50V_I54M_V82F	-2.755252485
M46I_V82T_I84V_L90M	-2.755403432
I47V_T74P_V82F_I84V	-2.755599193
V32I_M46I_I47V_I54L_V82T_I84V_L90M	-2.756810127
M46I_I50V_V82F_L90M	-2.757430639
V32I_V82T_L90M	-2.75931813
V32I_M46I_T74P_L76V	-2.760352958
M46I_I50V_L76V_V82T_L90M	-2.761892164
I47V_I54L_L76V_L90M	-2.763015739
V32I_M46I_I54L_V82F_L90M	-2.763951268

I47V_I54L_V82T_I84V_L90M	-2.76425258
V32I_I50V_L76V_V82F	-2.765055197
I47V_V82T_L90M	-2.766181523
V32I_M46I_I50V_I54L_L76V_V82F_I84V_L90M	-2.767488116
V32I_I47V_I54M_V82F_I84V	-2.77036278
V32I_I54M_I84V	-2.77082035
V32I_I50V_I54M_V82F_I84V_L90M	-2.771019043
V32I_M46I_I47V_I50V_I84V	-2.771163291
I47V_I54L_V82T	-2.771174343
V32I_M46I_I50V_I54M_V82F_I84V	-2.772706255
V32I_M46I_I84V_L90M	-2.772972176
V32I_M46I_I54M_V82F_I84V_L90M	-2.774320493
M46I_I47V_I50V_V82F_I84V_L90M	-2.775166798
I47V_I50V_I54L_L76V_V82T_I84V_L90M	-2.775166798
V32I_I54M_V82T_L90M	-2.775919179
V32I_I50V_I54L_V82T	-2.776623371
V32I_I54L_I84V_L90M	-2.77769548
V32I_I54L_V82F_I84V_L90M	-2.777710253
I47V_V82F_L90M	-2.778643649
M46I_I47V_L76V_V82F_L90M	-2.779375242
V32I_I50V_L76V_V82T	-2.78194754
I47V_I50V_L76V_V82T_I84V_L90M	-2.782095551
M46I_I54L_V82F_L90M	-2.782104783
M46I_I50V_I54L_I84V_L90M	-2.78253581
V32I_I54M_V82F_I84V_L90M	-2.782583198
V32I_I50V_I54L_V82F_I84V	-2.783255836
I54M_L76V_V82F_I84V_L90M	-2.783793986
V32I_M46I_I47V_I54M_L76V_V82T_L90M	-2.783878744
I54M_L76V_I84V_L90M	-2.785702411
M46I_I47V_I50V_I84V_L90M	-2.786285527
V32I_I50V_I54M_L90M	-2.788312074
V32I_M46I_I47V_I54M_V82F_I84V	-2.788610933
I50V_V82F_I84V	-2.789033107
V32I_I47V_I50V_I54L_V82F_I84V_L90M	-2.789638204
V32I_I50V_I54L_V82F_I84V_L90M	-2.790230495
V32I_I47V_I50V_I54M_L76V_V82T_L90M	-2.790521579
I50V_L76V_L90M	-2.79154817
V32I_I84V_L90M	-2.792120443
V32I_I47V_I54L_L76V_V82T_L90M	-2.792587685
M46I_I50V_I54M_L76V_V82F_I84V	-2.792640917
I47V_I50V_I54L_L76V_V82F_L90M	-2.793343731
M46I_I47V_V82F_L90M	-2.794284975
M46I_I47V_I54L_L76V_I84V	-2.794609897

I47V_I50V_V82F_L90M	-2.794976274
M46I_I47V_L76V_V82T_I84V_L90M	-2.795471195
V32I_M46I_I47V_I50V_I54L_L76V_V82F_I84V	-2.795581499
I47V_I54L_V82T_I84V	-2.795995558
I47V_I50V_L76V_V82F	-2.796043285
I50V_I54L_V82F_L90M	-2.796188852
V32I_I47V_I54M_L76V_V82F_I84V	-2.797094203
V32I_M46I_I47V_L76V_V82F	-2.798720006
M46I_I54L_V82F_I84V_L90M	-2.799494486
V32I_I47V_I50V_I84V_L90M	-2.799494486
V32I_M46I_I50V_I54L_I84V_L90M	-2.79950404
V32I_M46I_I50V_I54L_V82F_I84V	-2.800458781
V32I_I54M_L76V_I84V_L90M	-2.801400706
I47V_I54L_L76V_I84V_L90M	-2.804340107
M46I_I47V_I54M_L76V_V82T_I84V_L90M	-2.804611728
I47V_I54M_V82T_L90M	-2.805578187
V32I_M46I_V82T_I84V	-2.807350968
I47V_L76V_V82T_I84V	-2.807538239
V32I_I47V_L76V_V82F_I84V	-2.810302578
I54M_L76V_V82F_L90M	-2.811786619
V32I_M46I_L76V_V82T_L90M	-2.812038896
M46I_I50V_I54L_V82T_L90M	-2.812038896
M46I_I54L_L76V_V82T_I84V	-2.8152313
V32I_I47V_I50V_L76V_I84V	-2.815912165
V32I_I54M_I84V_L90M	-2.816933479
M46I_I50V_L76V_V82F	-2.817002137
L10F_I47V_T74P	-2.817208499
M46I_I47V_I50V_I54L_V82T_I84V_L90M	-2.817638991
M46I_I47V_I50V_L76V_V82T_I84V	-2.817700411
M46I_I47V_I54L_V82T_L90M	-2.818044037
V32I_I54M_V82F_I84V	-2.818605544
M46I_I54M_L76V_V82F_L90M	-2.820626208
V32I_M46I_I47V_T74P_V82F	-2.821198026
M46I_I47V_I54M_I84V_L90M	-2.821542879
M46I_I47V_I50V_I54L_V82F_I84V_L90M	-2.821852771
V32I_I54M_L90M	-2.822798568
V32I_M46I_I47V_I54L_V82F_L90M	-2.825184825
V32I_I54M_L76V_V82F	-2.825723103
V32I_M46I_I47V_T74P_L76V	-2.828838954
V32I_M46I_I47V_I54L_L76V_V82F_L90M	-2.830165915
V32I_M46I_I47V_I50V_I54L_L76V_I84V	-2.831864409
V32I_I50V_V82F_L90M	-2.833050173
V32I_I54L_L76V_V82F_I84V	-2.833052959

M46I_I47V_L76V_L90M	-2.833586822
V32I_I50V_I84V_L90M	-2.833963757
V32I_M46I_I47V_I54M_L76V_V82F_I84V_L90M	-2.834386778
L10F_V32I_T74P	-2.834442082
V32I_M46I_I47V_I54M_I84V_L90M	-2.834736416
V32I_M46I_I50V_V82F_I84V	-2.836078327
M46I_I50V_I54M_V82T_L90M	-2.837612878
V32I_I47V_I50V_V82T	-2.841260915
M46I_I54M_L76V_V82T_L90M	-2.841811338
V32I_I47V_I54L_T74P_V82F_I84V	-2.843099868
V32I_I47V_I50V_I54L_L76V_V82F_L90M	-2.844338519
I47V_I50V_I54L_V82T_L90M	-2.84621305
V32I_I47V_I50V_I54L_L76V_V82F_I84V_L90M	-2.846634965
I47V_I54L_L76V_V82T_I84V_L90M	-2.847731928
M46I_I47V_I50V_V82F_L90M	-2.848774364
M46I_L76V_I84V_L90M	-2.85204933
I47V_I54L_L76V_V82T_L90M	-2.853009306
I47V_I50V_I54L_L76V_V82F	-2.854258065
V32I_M46I_I47V_I54M_V82T_I84V_L90M	-2.854292014
M46I_I47V_I50V_L90M	-2.85448195
I50V_I54L_V82T_I84V_L90M	-2.855327282
V32I_M46I_I47V_I50V_I54L_I84V_L90M	-2.85673071
M46I_I54L_L76V_V82F_I84V_L90M	-2.85673071
M46I_I50V_V82T_I84V_L90M	-2.85673071
V32I_M46I_I50V_I54M_L76V_V82F_L90M	-2.85673071
V32I_M46I_I47V_I54L_L76V_L90M	-2.856819709
V32I_I50V_I54L_I84V_L90M	-2.857894065
M46I_I47V_I54L_L76V_L90M	-2.860024969
M46I_I47V_I54M_V82F_I84V_L90M	-2.861859827
I47V_V82T_I84V_L90M	-2.863434375
I50V_L76V_I84V	-2.864722617
V32I_M46I_I50V_L76V_V82T	-2.865712708
V32I_I50V_I54L_V82T_I84V	-2.866305969
V32I_M46I_I47V_L76V_L90M	-2.866886362
M46I_I47V_I54L_L76V_I84V_L90M	-2.869753618
V32I_M46I_I54L_L76V_V82F_L90M	-2.871036634
I47V_I54M_V82F_I84V_L90M	-2.871565275
V32I_I47V_I54L_L76V_I84V	-2.874292049
V32I_I47V_I50V_I54L_L76V_V82F_I84V	-2.874605418
V32I_M46I_I54L_L76V_L90M	-2.876196794
I47V_I50V_I54L_V82T_I84V	-2.878744795
M46I_I54M_L76V_I84V_L90M	-2.881819735
I47V_I50V_I84V_L90M	-2.881882498

V32I_M46I_I50V_I54L_I84V	-2.882841838
L10F_I47V_V82F	-2.885759791
M46I_I50V_I54L_V82F	-2.886137456
L10F_V32I_I47V_V82F	-2.887573186
M46I_I47V_I54M_V82T_L90M	-2.888366517
I47V_I50V_I54L_L76V_V82T_L90M	-2.888763788
V32I_I50V_V82T	-2.889830248
M46I_I50V_I54L_L76V_V82F_I84V	-2.891840254
I47V_L76V_L90M	-2.892050675
M46I_I47V_L76V_V82T_L90M	-2.89413112
I47V_I50V_L76V_V82T_L90M	-2.89451773
V32I_I47V_I50V_I54M_V82T_I84V	-2.897451109
I54L_L76V_V82T_I84V_L90M	-2.898456637
M46I_L76V_L90M	-2.899474971
I47V_I50V_I54M_V82F_I84V_L90M	-2.901391673
M46I_I50V_I54L_V82F_L90M	-2.902528985
I50V_I54L_L76V_V82T_L90M	-2.909494886
V32I_I47V_I50V_I54M_V82F_I84V_L90M	-2.909494886
M46I_I50V_I84V_L90M	-2.912033518
I47V_I50V_I54L_L76V_L90M	-2.91350245
I47V_I54M_L76V_L90M	-2.913588074
I47V_I50V_L76V_V82F_L90M	-2.914440045
V32I_I54L_L76V_V82T_I84V_L90M	-2.914440045
M46I_I47V_I50V_I54L_L76V_V82F_L90M	-2.915473365
I50V_V82F_L90M	-2.91618681
V32I_I47V_I50V_V82F_I84V_L90M	-2.916794124
I47V_I50V_I54L_L90M	-2.919795708
V32I_M46I_I47V_I54L_I84V_L90M	-2.920159203
V32I_M46I_I47V_I54M_V82T_L90M	-2.920275247
V32I_M46I_I50V_I54L_L76V_I84V	-2.921306311
V32I_I47V_I54M_L76V_V82F_L90M	-2.924220277
V32I_M46I_I54M_L76V_V82T_L90M	-2.925969296
V32I_V82T_I84V_L90M	-2.926315803
M46I_I47V_I50V_I54L_V82F_L90M	-2.926650706
I50V_I54L_L76V_V82F_L90M	-2.928300485
V32I_I50V_V82T_I84V	-2.929348222
I47V_I50V_I54L_V82F_I84V_L90M	-2.930314889
V32I_M46I_L76V_L90M	-2.930512184
V32I_M46I_I47V_I84V_L90M	-2.930767442
V32I_L76V_V82F_I84V_L90M	-2.931322301
V32I_I54M_V82T_I84V_L90M	-2.93168591
V32I_I50V_I54L_L76V_V82F	-2.932172194
L10F_T74P	-2.93257316

I50V_V82T_L90M	-2.933296637
M46I_I47V_I50V_I54M_L76V_V82F	-2.935110164
M46I_I54L_L76V_L90M	-2.936265004
V32I_L76V_I84V_L90M	-2.937386958
V32I_I50V_V82T_L90M	-2.944121959
L10F_V32I_V82F	-2.944948233
V32I_I47V_I54L_L76V_V82F_I84V_L90M	-2.949210554
V32I_I47V_I50V_L76V_V82F_I84V	-2.949799739
V32I_M46I_I54L_L76V_V82T_I84V_L90M	-2.949799739
L10F_V82F	-2.952585865
V32I_I47V_I50V_I54M_L76V_V82T	-2.952672702
V32I_V82F_I84V_L90M	-2.953355249
V32I_I47V_I54L_L76V_V82F_L90M	-2.956161912
V32I_M46I_I50V_V82F_I84V_L90M	-2.956845863
I47V_I54L_L76V_V82F_L90M	-2.957205686
I47V_I54L_V82F_I84V_L90M	-2.958304273
V32I_M46I_I47V_I50V_L76V_V82F_I84V_L90M	-2.958304273
V32I_I47V_L76V_V82T_I84V_L90M	-2.961082916
V32I_I47V_V82F_I84V_L90M	-2.961705783
V32I_I50V_I54M_I84V_L90M	-2.961705783
V32I_I47V_I54L_V82T_I84V_L90M	-2.964403333
V32I_M46I_I47V_I50V_I54L_V82T_I84V_L90M	-2.96527903
I47V_I50V_V82T_L90M	-2.965812221
V32I_M46I_L76V_V82T	-2.967198724
V32I_I50V_I54L_L76V_L90M	-2.967538841
V32I_M46I_I54M_V82F_L90M	-2.96827681
I47V_I54M_L76V_V82T_I84V_L90M	-2.968460911
M46I_L76V_V82F_L90M	-2.968842107
V32I_M46I_I47V_L76V_V82F_I84V	-2.969358578
V32I_I47V_I50V_I54L_V82T_I84V_L90M	-2.96943499
V32I_I47V_I50V_L76V_V82F_L90M	-2.971523553
V32I_M46I_I50V_I54L_L76V_V82T_I84V	-2.973613286
V32I_L76V_V82F_I84V	-2.974307644
M46I_I47V_I54M_V82F_L90M	-2.974997983
V32I_L76V_V82T_L90M	-2.975581131
I47V_I50V_I54L_V82F_L90M	-2.975643652
V32I_I54L_L76V_V82F_I84V_L90M	-2.976143881
V32I_I47V_I54M_L76V_V82T_I84V_L90M	-2.977092043
M46I_I54L_L76V_V82F_L90M	-2.977491965
I50V_L76V_V82F_I84V_L90M	-2.977629468
V32I_M46I_I47V_I54L_L76V_V82F_I84V_L90M	-2.983116582
V32I_I54M_V82F_L90M	-2.983740914
V32I_M46I_I47V_I54L_V82F_I84V_L90M	-2.986466275

V32I_M46I_I54M_L76V_L90M	-2.989638376
V32I_I47V_L76V_V82T_L90M	-2.991505971
M46I_I47V_I54L_V82F_L90M	-2.992326002
V32I_I47V_I54L_V82F_L90M	-2.994754109
V32I_M46I_I50V_I54L_V82F_L90M	-2.995070691
V32I_I47V_I50V_I54M_L76V_V82F_I84V_L90M	-2.995070691
M46I_I47V_V82T_I84V_L90M	-3.002118488
V32I_I54L_V82F_L90M	-3.0073194
V32I_I47V_I54L_I84V_L90M	-3.008063425
L10F_V32I_T74P_V82F	-3.00843883
V32I_I54M_L76V_V82F_L90M	-3.009884011
I47V_I50V_L76V_L90M	-3.011231501
V32I_M46I_I50V_I54L_L90M	-3.01270368
V32I_M46I_I47V_I50V_I54L_L76V_V82T_I84V_L90M	-3.01270368
I47V_I50V_V82T_I84V_L90M	-3.015784299
V32I_I47V_I54M_V82F_I84V_L90M	-3.019456466
I50V_V82F_I84V_L90M	-3.019624045
V32I_M46I_I47V_I50V_L76V_L90M	-3.020127698
V32I_I47V_V82T_L90M	-3.022150318
I47V_I54L_V82T_L90M	-3.02577053
I54L_V82F_I84V_L90M	-3.028088441
M46I_I47V_I54M_L76V_I84V_L90M	-3.034605521
V32I_M46I_I47V_L76V_V82F_I84V_L90M	-3.034605521
I47V_I54L_L76V_V82F_I84V_L90M	-3.035229313
V32I_I47V_I54M_L76V_V82F_I84V_L90M	-3.037456063
V32I_I47V_I54M_I84V_L90M	-3.039941832
I47V_I50V_L76V_I84V_L90M	-3.040140351
V32I_I47V_V82F_L90M	-3.040890863
V32I_I47V_I50V_I54L_V82T_L90M	-3.041667376
V32I_I47V_I54L_L76V_V82T	-3.046813137
I50V_L76V_V82T_L90M	-3.048616236
M46I_I47V_I54M_L76V_V82F_L90M	-3.059672932
I47V_V82F_I84V_L90M	-3.061066858
V32I_M46I_V82F_L90M	-3.064048986
V32I_I54L_L76V_I84V	-3.067381112
V32I_I54M_L76V_L90M	-3.075350489
M46I_I54M_L76V_V82F_I84V_L90M	-3.083001887
M46I_I50V_I54M_L76V_L90M	-3.083001887
M46I_I47V_I54L_V82F_I84V_L90M	-3.085545277
V32I_I54M_L76V_V82T_L90M	-3.092568227
I47V_I54M_V82T_I84V_L90M	-3.092662305
V32I_I54L_V82T_I84V_L90M	-3.092939102
V32I_I50V_L76V_L90M	-3.094373726

V32I_M46I_I47V_I50V_V82F_L90M	-3.097991882
V32I_M46I_I54L_L76V_V82T_L90M	-3.106988055
V32I_I47V_I54L_V82T_L90M	-3.110262449
V32I_I47V_L76V_V82F_I84V_L90M	-3.115532432
I47V_I50V_V82F_I84V_L90M	-3.117467829
V32I_I47V_I50V_V82F_L90M	-3.118730406
V32I_I47V_L76V_I84V_L90M	-3.122228022
M46I_I47V_I54L_L76V_V82F_L90M	-3.126066445
I47V_L76V_V82T_L90M	-3.12986895
V32I_I47V_L76V_L90M	-3.138057181
I54L_L76V_V82F_I84V_L90M	-3.176132395
V32I_M46I_I47V_L76V_V82F_L90M	-3.181241801
V32I_L76V_L90M	-3.182357104
V32I_V82F_L90M	-3.232583435
V32I_I47V_I54L_V82F_I84V_L90M	-3.243090089
V32I_I54L_V82T_L90M	-3.265504336
L10F_T74P_V82F	-3.338735946
