# Supplementary material for: Predominance of positive epistasis among drug resistance-associated mutations in HIV-1 protease
Source: PLoS Genet. 2020 Oct 21;16(10):e1009009. doi: 10.1371/journal.pgen.1009009 (PMC7605711; doi:10.1371/journal.pgen.1009009)
Supplement: S2 Table — (PDF) [file pgen.1009009.s010.pdf]

Supplementary Table 2: Information of protease inhibitor resistance associated mutations covered in the library.

mutant	average relative fitness	distance to active center (Å)	TPV resistance score[a]	DRV resistance score[a]	relative fitness in reference[b]
L10F	-0.91	11.290	0	5	0.00
V32I	-1.70	12.490	5	15	-0.67
M46I	-0.25	19.092	5	0	-0.06
I47V	-0.79	15.447	30	10	-0.20
I50V	-2.25	12.750	-5	20	-0.81
I54L	-1.06	18.017	-10	20	-0.10
I54M	-1.28	18.017	20	20	-0.11
T74P	-2.03	17.498	25	5	-0.43
L76V	-2.03	16.394	-5	20	-0.84
V82T	-1.03	12.256	45	0	-0.38
V82F	-2.33	12.256	0	15	-0.31
I84V	-0.87	8.741	30	15	-0.20
L90M	-0.19	10.462	0	0	0.09

[a] From Stanford Drug Resistance Database's curation.

[b] Boucher JI, Whitfield TW, Dauphin A, Nachum G, Hollins III C, Zeldovich KB, et al. Constrained mutational sampling of amino acids in HIV-1 protease evolution. Molecular biology and evolution. 2019;36(4):798–810.
