# Supplementary material for: Predominance of positive epistasis among drug resistance-associated mutations in HIV-1 protease
Source: PLoS Genet. 2020 Oct 21;16(10):e1009009. doi: 10.1371/journal.pgen.1009009 (PMC7605711; doi:10.1371/journal.pgen.1009009)
Supplement: S3 Table — (PDF) [file pgen.1009009.s011.pdf]

Supplementary Table 3: Sequence of oligonucleotides used in this research.

oligonucleotides	sequence
O1F	ACTCTTTGGCAGCGACCC**YTC**GTCACAATAAAGATAGGGGGGCAATTAAAGGAAGCT
O1R	/5Phos/TCCTTTAATTGCCCCCCTATCTTTATTGTGAC**GAR**GGGTCGCTGCCAAAGAGT
O2F	/5Phos/CTATTAGATACAGGAGCAGATGATACA**RTA**TTAGAAGAAATGAATTTGCCAGGA
O2R	TCCTGGCAAATTCATTTCTTCTAA**TAY**TGTATCATCTGCTCCTGTATCTAATAGAGCT
O3F1	TGGAAACCAAAA**ATSRTA**GGGGGA**RTT**GGAGGTTTT**ATC**AAAGTAAGACAGTATGAT
O3F2	TGGAAACCAAAA**ATSRTA**GGGGGA**RTT**GGAGGTTTT**ATG**AAAGTAAGACAGTATGAT
O3F3	TGGAAACCAAAA**ATSRTA**GGGGGA**RTT**GGAGGTTTT**CTC**AAAGTAAGACAGTATGAT
O3R1	/5Phos/TACTGTCTTACTTT**GAT**AAAACCTCC**AAY**TCCCCC**TAYSAT**TTTTGGTTTCCA
O3R2	/5Phos/TACTGTCTTACTTT**GAT**AAAACCTCC**AAY**TCCCCC**TAYSAT**TTTTGGTTTCCA
O3R3	/5Phos/TACTGTCTTACTTT**GAT**AAAACCTCC**AAY**TCCCCC**TAYSAT**TTTTGGTTTCCA
O4F	/5Phos/CAGATACTCATAGAAATCTGCGGACATAAAGCTATAGGT**MCA**GTA**KTA**GTAGGA
O4R	TCCTAC**TAM**TAC**TGK**ACCTATAGCTTTATGTCCGCAGATTTCTATGAGTATCTGATCA
O5F1	/5Phos/GACCTACACCT**GTC**AAC**RTA**ATTGGAAGAAATCTG**WTG**ACTCAGATTGGCTGCACTTTA
O5F2	/5Phos/GACCTACACCT**ACC**AAC**RTA**ATTGGAAGAAATCTG**WTG**ACTCAGATTGGCTGCACTTTA
O5F3	/5Phos/GACCTACACCT**TTC**AAC**RTA**ATTGGAAGAAATCTG**WTG**ACTCAGATTGGCTGCACTTTA
O5R1	TAAAGTGCAGCCAATCTGAGT**CAW**CAGATTTCTTCCAAT**TAY**GTT**GAC**AGGTGTA
O5R2	TAAAGTGCAGCCAATCTGAGT**CAW**CAGATTTCTTCCAAT**TAY**GTT**GAC**AGGTGTA
O5R3	TAAAGTGCAGCCAATCTGAGT**CAW**CAGATTTCTTCCAAT**TAY**GTT**GAC**AGGTGTA
Recover_O1+O2_F	ACTCTTTGGCAGCGACCC
Recover_O1+O2_R	CCGGTCTCTCTTCCTGGCAAATTCATTTCTTC
Recover_O3+O4_F	TGGAAACCAAAAATSRTAGGG
Recover_O3+O4_R	CCGGTCTCAGGTCCTACTAMTACTGKACCTATAGC
Recover_O345_F	CCGGTCTCGGAAGATGGAAACCAAAAATSRTAGGG
Recover_O345_R	TAAAGTGCAGCCAATCTGAGT
Up_ApaI_F	GCAGGGCCCCTAGGAAA
Up_R	GGGTCGCTGCCAAAGAGT
Down_F	ACTCAGATTGGCTGCACTTTA
Down_SbfI_R	AACCCTGCAGGATGTGG
Mut1_L90M_F	GAAGAAATCTGATGACTCAGATTGG
Mut2_V82T_F	CACCTACCAACATAATTGGAAGAAATC
Mut3_V82F_F	CACCTTTCAACATAATTGGAAGAAATC
Mut4_L76V_F	CTATAGGTACAGTAGTAGTAGGACCTAC
Mut5_T74P_F	CTATAGGTCCAGTATTAGTAGGACCTAC
Mut6_I47V_F	GGAAACCAAAAATGGTAGGGGGAATTG
Mut7_L10F_F	CGACCCTTCGTCACAATAAAG
Mut1_L90M_R	CCAATCTGAGTCATCAGATTTCTTC
Mut2_V82T_R	GATTTCTTCCAATTATGTTGGTAGGTG
Mut3_V82F_R	GATTTCTTCCAATTATGTTGAAAGGTG
Mut4_L76V_R	GTAGGTCCTACTACTACTGTACCTATAG
Mut5_T74P_R	GTAGGTCCTACTAATACTGGACCTATAG
Mut6_I47V_R	CAATTCCCCCTACCATTTTTGGTTTCC
Mut7_L10F_R	CTTTATTGTGACGAAGGGTCG
